# The synthesis of novel thioderivative chalcones and their influence on NF-κB, STAT3 and NRF2 signaling pathways in colorectal cancer cells

**DOI:** 10.1038/s41598-022-18981-4

**Published:** 2022-09-01

**Authors:** Katarzyna Papierska, Violetta Krajka-Kuźniak, Robert Kleszcz, Tomasz Stefański, Rafał Kurczab, Maciej Kubicki

**Affiliations:** 1 Department of Pharmaceutical Biochemistry, Poznan University of Medical Sciences, Święcickiego 4, 60-781 Poznań, Poland; 2 Faculty of Chemistry, Adam Mickiewicz University in Poznań, Uniwersytetu Poznańskiego 8, 61-712 Poznań, Poland; 3 Department of Medicinal Chemistry, Maj Institute of Pharmacology, Polish Academy of Sciences, Smetna 12, 31-343 Krakow, Poland

**Keywords:** Biochemistry, Cancer, Cell biology

## Abstract

This study aimed to synthesize new thioderivative chalcones and analyze their impact on the NF-κB, STAT3, EGFR and Nrf2 signaling pathways in colorectal cancer cells. Among the studied compounds, derivatives **4** and **5** decreased the activation of NF-κB and the expression of the target gene COX-2. In the case of STAT3, we observed the inhibition of activation of this signaling pathway after influencing derivative **4**. Increased activation of the Nrf2 signaling pathway was demonstrated for derivatives **5** and **7** in DLD-1 and HCT116 cells. The results of this study indicated that new chalcone derivatives, especially compounds **4, 5,** and—to some degree—**7,** possess potential applications in the prevention of colorectal cancer.

## Introduction

Colorectal cancer (CRC) is the third most deadly and fourth most commonly diagnosed cancer in the world^1^. As a result, continuous advances in the treatment and prevention of CRC are expected, including the use of natural or synthetic compounds that alter the transduction of signals in normal and cancer cells. Essential features of carcinogenesis include dysregulation of signaling pathways, proinflammatory signaling, and inactivation of the products of oxidative stress, which are features that are especially relevant in colorectal cancer^2^. Inflammation plays a crucial role in the development of colorectal carcinogenesis. Inflammatory cytokines activate the nuclear factor-κB (NF-κB) signaling pathway in tumor cells and inflammatory cells^3^. Recent studies showed that EGFR signaling is involved in inflammatory response and is also a known target of NF-κB. The EGFR is a transmembrane protein of the ErbB tyrosine kinases family and when it is activated, multiple signaling pathways are induced, thus modulating pleiotropic cell responses, such as proliferation, migration and apoptosis^4^.

In addition, the classic signal transducer and activator of transcription (STAT3) signaling pathway is also activated by inflammatory cytokines. The STAT3 transcription factor then translocates to the nucleus and controls the transcription of several apoptotic and cell cycle regulatory proteins^5^.

The Kelch-like ECH-associated protein 1 (Keap1)-NF-E2 related factor (Nrf2) system regulates the expression of a battery of cytoprotective genes in response to electrophilic and oxidative stresses. Nrf2, a key transcription factor, regulates the basal and inducible expression of numerous detoxifying and antioxidant genes (e.g., NQO1, GST) and stress-response proteins (e.g., SOD)^6^.

Chalcones, derivatives of (E)-1,3-diphenyl-2-propene-1-one, are being widely studied due to their multidirectional mechanism of anticancer action (cf. for instance, some recent reviews and references therein)^7–10^. In particular, there were reports of methoxy-derivatives of chalcones which activities are targeting the NF-κB cell signaling pathway^11^. In particular, it has been found that the anticancer activity of 3-hydroxy-4,3’,4’,5’-tetramethioxychalcone correlates with its NF-κB inhibitory activity. Similar correlations have been found for Nrf2^12^ as well as for STAT3 signaling pathways^13^.

**Scheme 1.**
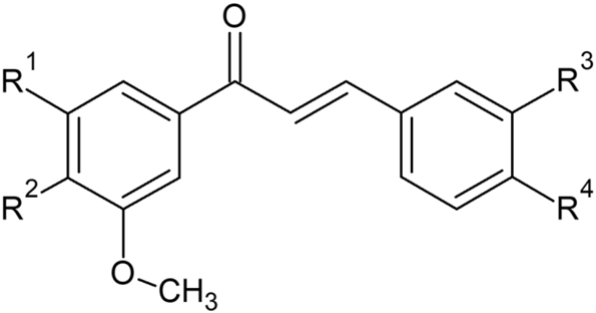
Molecular structures of studied chalcones. **1**: R^1^ = R^2^ = R^4^ = OMe, R^3^ = SMe; **2**: R^1^ = Br, R^2^ = R^3^ = OMe, R^4^ = SMe; **3**: R^1^ = Br, R^2^ = R^4^ = OMe, R^3^ = SMe; **4**: R^1^ = Br, R^2^ = OMe, R^3^ = SMe, R^4^ = H; **5**: R^1^ = Br, R^2^ = R^4^ = SMe, R^3^ = OMe; **6**: R^1^ = Br, R^2^ = R^3^ = SMe, R^4^ = OMe; **7**: R^1^ = Br, R^2^ = R^4^ = SMe, R^3^ = H; **8**: R^1^ = R^4^ = OMe, R^2^ = R^3^ = SMe.

Relatively simple molecular structure makes chalcones a very promising object for modifications which creates a chance for the development of new, effective chemotherapeutics and for the better understanding, at the molecular level, of their interaction with the appropriate binding site. The bioisosteric replacements have been found to increase significantly the anticancer activity of these compounds, for instance, H to F isosteric replacement^14^. In the previous studies of CA-4 analogues, it was found that the replacement of methoxy into methylthio group may also increase the specifical anticancer actions^15^. Therefore, we decided to synthesize and scrutinize a number of thiomethoxy derivatives with chalcone skeleton (Scheme 1).

Current research focusing on therapeutic targets represents a novel trend in cancer therapy. This novel trend is known as *anakoinosis* and is based on the mutual communication of signaling pathways in cancer cells^16^. The transcription factors Nrf2, NF-κB, and STAT3, which are overexpressed in CRC cells, play an important role in colon carcinogenesis. Therefore, new synthetic derivatives of chalcones could be used both in the prophylaxis and treatment of CRC.

Therefore, this study aimed to evaluate the influence on the Nrf2, NF-κB, EGFR, and STAT3 signaling pathways and their target genes in connection with cell cycle distribution, apoptosis, and migration in colon cancer cell lines. In addition, we applied an ADME analysis and molecular docking analysis to reveal the possible mechanisms of the selected chalcone’s anti-tumor effect.

## Results

### The effect of thioderivative chalcones on the viability of DLD-1 and HCT116 cells

**Figure 1:**
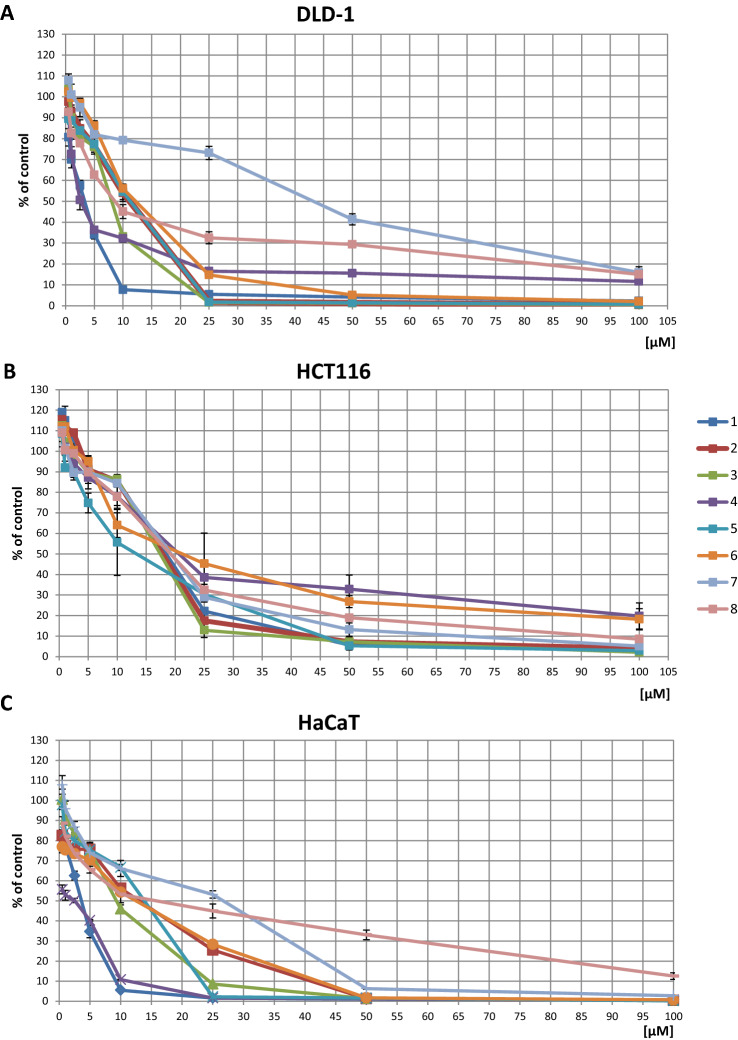
The effect of thioderivative chalcones on the viability of DLD-1 (**A**), HCT116 (**B**) and HaCaT **(C)** cells. Mean values ± SEM from three independent experiments are shown.

The cytotoxicity of new chalcones in DLD-1 and HCT116 cells was evaluated in the range of 0.5–100 µM (Fig. 1A,B). The IC50 values determined in DLD-1 cells for chalcones **1, 2, 3, 4, 5, 6, 7,** and **8** (Scheme 1) were 3, 11, 8, 2, 11, 12, 43, and 9 μM, respectively. The IC50 values determined in HCT116 cells for chalcones **1, 2, 3, 4, 5, 6, 7,** and **8** were 19, 18, 17, 20, 13, 21, 19, and 19 μM, respectively. In DLD-1 cells, compound **4** showed the highest cytotoxicity, while compound **7** showed the lowest cytotoxicity. In HCT116 cells, no significant differences in IC50 values were found for the tested chalcones. Based on the MTT test results, chalcone concentrations ensuring at least 70% cell survival were selected for further studies. Therefore, to evaluate the activity and expression of the tested parameters, the following concentrations of thioderivative chalcones were selected: chalcone **1** at 1 μM (1/1); chalcone **2** at 5 μM (2/5); chalcone **3** at 5 μM (3/5); chalcone **4** at 1 μM (4/1); chalcone **5** at 5 μM (5/5); chalcone **6** at 5 μM (6/5); chalcones **7** at 5 μM (**7**/5); chalcones **8** at 5 μM (8/5).

In order to verify whether the cytotoxic effects of the studied chalcones are specific towards colon cancer cells, we estimated their impact on the normal human HaCaT cells (Fig. 1C). The IC50 values determined for this cell line for chalcones **1–8** were 4, 13, 9, 3, 14, 13, 27 and 17 μM, respectively (Fig. 1C).

### The effect of thioderivative chalcones on the activation and expression of NF-κB and target genes

**Figure 2:**
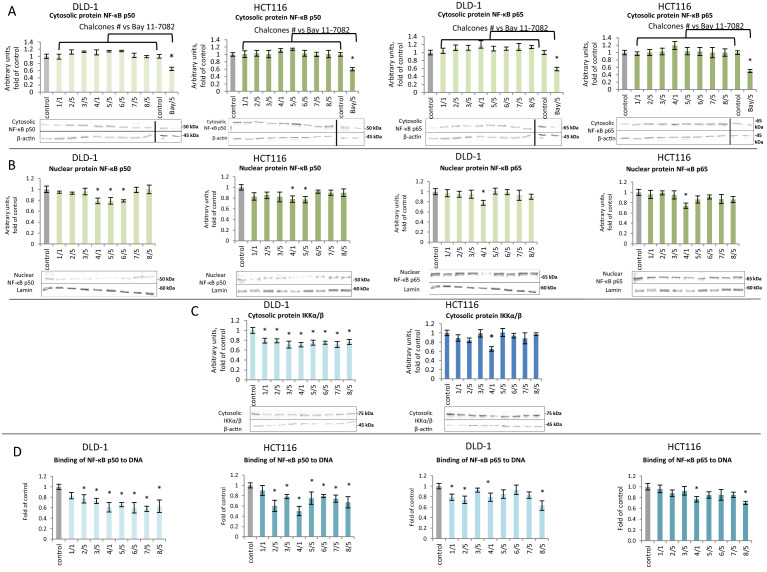
The effect of synthetic thioderivative chalcones compounds and Bay 11-7082 on the level of NF-κB activation in DLD-1 and HCT116 cells. (**A)** The levels of NF-κB, p50, and p65 protein in the cytosolic fraction. (**B) **The levels of NF-κB p50 and p65 protein in the nuclear fraction. (**C)** The levels IKKα/β protein in the cytosolic fraction. Representative Western immunoblots are presented under the graphs (**A–C**). (**D**) The level of NF-κB, p50, and p65 binding to DNA. The values (mean ± SEM) were calculated as a relative change in transcript or protein level in comparison to control cells (where control expression equals 1). The values from three separate experiments run in triplicate are presented. Asterisk: mean values were significantly different from the control group (p ≤ 0.05). Hash: mean values were significantly different from the Bay 11–7082 treated group (p ≤ 0.05). Original scans of blots are shown in Supplementary Fig. S1.

The effect of a thioderivative chalcones on the activation of transcription factor NF-κB was assessed based on translocation and binding to the target sequences of the active subunits of the p50/p65 dimer (Fig. 2A,B). The use of chalcones **4** and **5** resulted in a significant 21% to 23% reduction in the level of the p50 subunit in the nuclear fraction of cells of both tested lines; the use of chalcone **6** yielded a similar result in DLD-1 cells (Fig. 2B). A similar pattern of changes was observed in the level of the p65 subunit. There was no significant change in the level of p65 in the cytosolic fraction. In contrast, in the nuclear fraction, the level of p65 decreased under the influence of chalcone **4** by 22% in DLD-1 cells and 26% in HCT116 cells (Fig. 2B).

Additionally, we found the inactivation of IKKα/β by all tested chalcones in DLD-1, whereas by compound **4** in HCT116 (Fig. 2C). This correlated with the suppression of the nuclear translocation of subunits p65 and p50.

Analysis of the effect of the chalcones on the binding of NF-κB subunits to the 5'-GGGACTTTCC-3' target sequence in DLD-1 cells revealed that all examined chalcones, except for compound **1**, decreased p50 binding, and that compound **7** decreased p50 binding to the greatest extent (41%) in relation to the control group (Fig. 2D). Compounds **1, 2, 4**, and **8** reduced the binding of the p65 subunit to DNA by 21%, 24%, 21%, and 38%, respectively (Fig. 2D).

**Figure 3:**
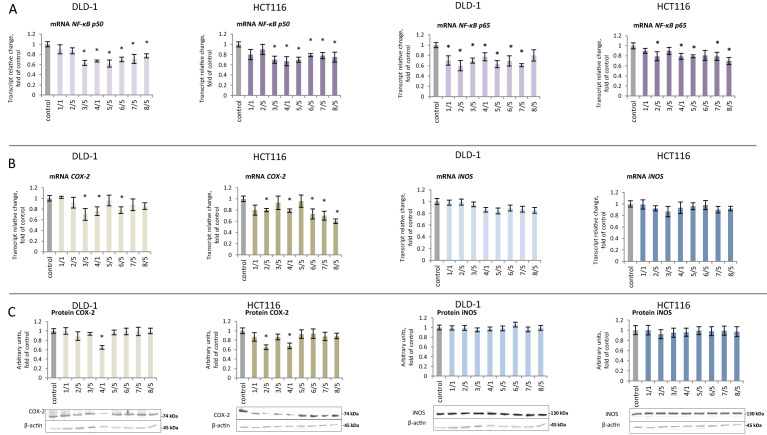
The effect of synthetic thioderivative chalcones compounds on the level of NF-κB expression in DLD-1 and HCT116 cells. (**A) **The levels of *NF-κB, p50,* and *p65* mRNA. (**B)** The level of mRNA of the selected target genes of NF-κB: *COX-2* and *iNOS*. (**C) **The levels of COX-2 and iNOS proteins in the cytosolic fraction. Representative Western immunoblots are presented under the graphs (**A–C**). The values (mean ± SEM) were calculated as a relative change in transcript or protein level in comparison to control cells (where control expression equals 1). The values from three separate experiments run in triplicate are presented. Asterisk: mean values were significantly different from the control group (p ≤ 0.05). Original scans of blots are shown in Supplementary Fig. S2.

Activation of NF-κB is the result of the release of active subunits from the cytoplasmic complex with IκB and the induction of the expression of genes encoding these subunits. Transcription of the *p50 NF-ĸB* subunit decreased 22–28% with the use of compounds **3–8** in both DLD-1 and HCT116 cells (Fig. 3A). The most potent inhibitory effect on the *p65* subunit was found for derivatives **2, 5** and **7** in DLD-1 cells. In contrast, in HCT116 cells, *p65* transcription was decreased to the greatest extent (30%) by the influence of compound **8**, as compared to the control group.

In DLD-1 cells, the use of chalcones **3, 4,** and **6** resulted in a statistically significant reduction in *COX-2* transcription (Fig. 3B). However, the decreased expression of *COX-2* could only be confirmed, via reduced COX-2 protein levels, in the case of compound **4** (Fig. 3C). In HCT116 cells, reduction of COX-2 expression was confirmed at both the transcript and protein levels due to the use of compounds **2** and **4** (Fig. 3B,C).

The *iNOS* gene expression analysis showed no significant differences in either the mRNA or protein levels of iNOS due to the use of the chalcones in either cell line (Fig. 3B,C).

In our study, we used BAY 11-7082 NF-κB inhibitor (5 µM) to compare the chalcone’s action on levels of NF-κBp65, NF-κBp50 and COX-2. In both tested cell lines, we observed a more potent inhibition of these parameters after the inhibitor treatment than under the influence of the studied chalcones (Fig. 2A).

### The effect of thioderivative chalcones on the activation and expression of STAT3 and target genes

**Figure 4:**
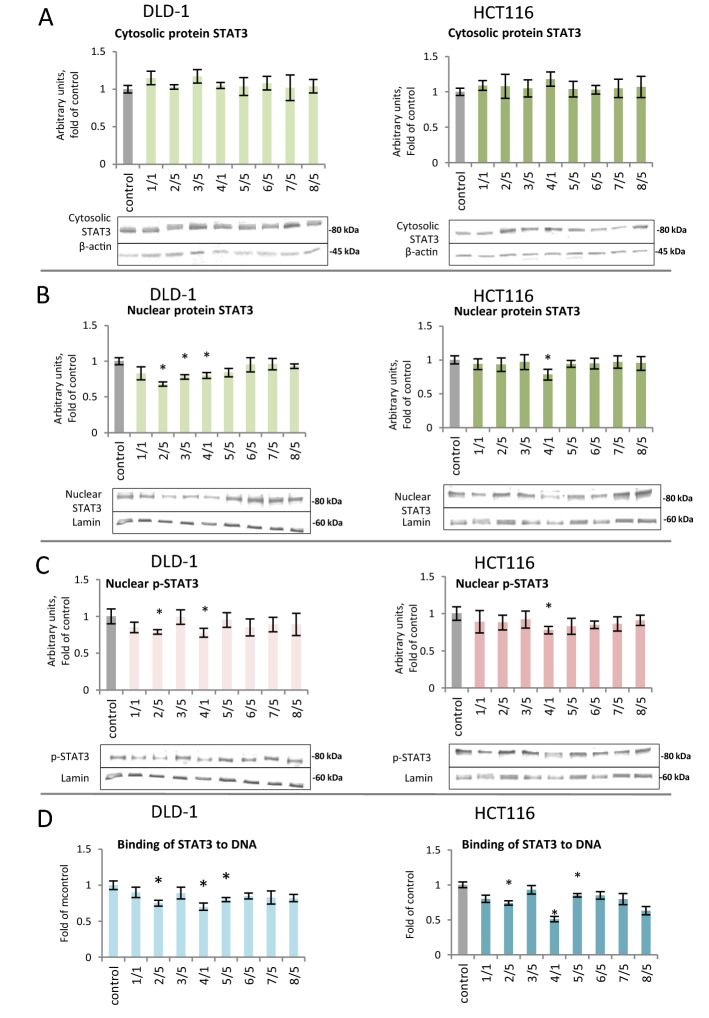
The effect of synthetic thioderivative chalcones compounds on the level of STAT3 activation in DLD-1 and HCT116 cells. (**A) **The level of STAT3 protein in the cytosolic fraction. (**B) **The level of STAT3 protein in the nuclear fraction. (**C) **The level of p-STAT3 protein in the nuclear fraction. Representative Western immunoblots are presented under the graphs (**A–C**). (**D)** The level of STAT3 binding to DNA. The values (mean ± SEM) were calculated as a relative change in transcript or protein level in comparison to control cells (where control expression equals 1). The values from three separate experiments run in triplicate are presented. Asterisk: mean values were significantly different from the control group (p ≤ 0.05). Original scans of blots are shown in Supplementary Fig. S3.

The Western blot of STAT3 protein levels in cytosolic and nuclear fractions revealed a significant decrease in the nuclear levels of STAT3 protein after treatment with chalcones **2** and **4,** by 22% and 21%, respectively, in the DLD-1 cell line (Fig. 4A,B); in the HCT116 line, the use of chalcone **4** yielded a decrease of 22% (Fig. 4B). In the nuclear fraction of DLD-1 cells, a decrease in the binding of STAT3 to DNA was observed under the influence of compounds **2** (25%)**, 4** (30%)**,** and** 5** (20%) (Fig. 4D). In HCT116 cells, the same compounds also decreased the binding of STAT3 to DNA. The p-STAT3 protein level analysis showed statistically significant differences in the cells of both examined colorectal carcinomas and in DLD-1 cells under the influence of chalcones **2** and **4** (Fig. 4C). A decrease of 21% to 23% in p-STAT3 protein expression was observed after incubation with these chalcones.

**Figure 5:**
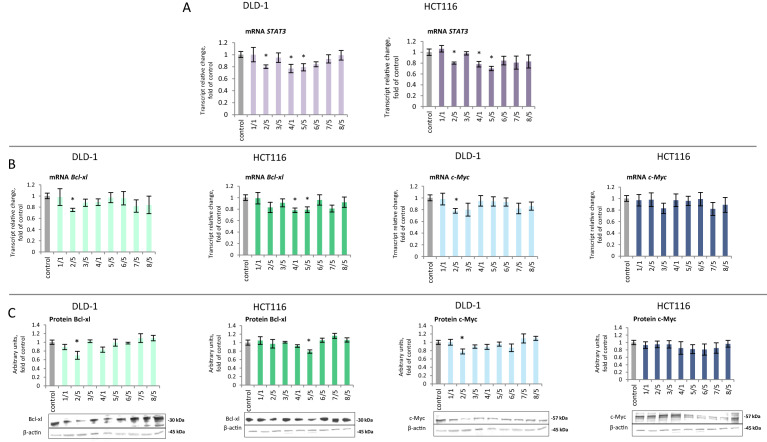
The effect of synthetic thioderivative chalcones compounds on STAT3 expression in DLD-1 and HCT116 cells. (**A)** The level of *STAT3* mRNA. (**B)** The level of mRNA of the selected target genes of STAT3: *Bcl-xl* and *c-Myc*. (**C) **The levels of Bcl-xl and c-Myc protein in the cytosolic fraction. Representative Western immunoblots are presented under the graphs (**A–C**). The values (mean ± SEM) were calculated as a relative change in transcript or protein level in comparison with control cells (where control expression equals 1). The values from three separate experiments run in triplicate are presented. Asterisk: mean values were significantly different from the control group (p ≤ 0.05). Original scans of blots are shown in Supplementary Fig. S4.

*STAT3* gene expression decreased in DLD-1 cells under the influence of thioderivative chalcones **2, 4, 5.** In HCT116 cells, a similar but slightly greater effect was also observed under the influence of the same compounds (Fig. 5A). A significant decrease in the level of *Bcl-xl* mRNA (25%) was noted in DLD-1 cells due to treatment with chalcone derivative **2**. Decreased *Bcl-xl* expression caused by this compound was also confirmed by a reduced level of the Bcl-xl protein. In HCT116 cells, compounds **4** and **5** decreased *Bcl-xl* transcription, but only for compounds **5,** it correlated with Bcl-xl protein level (Fig. 5B,C). In DLD-1 cells, chalcone **2** decreased c-Myc expression. However, none of the tested compounds significantly altered the expression of *c-Myc* in HCT116 cells (Fig. 5B,C).

### The effect of thioderivative chalcones on the activation and expression of Nrf2 and target genes

**Figure 6:**
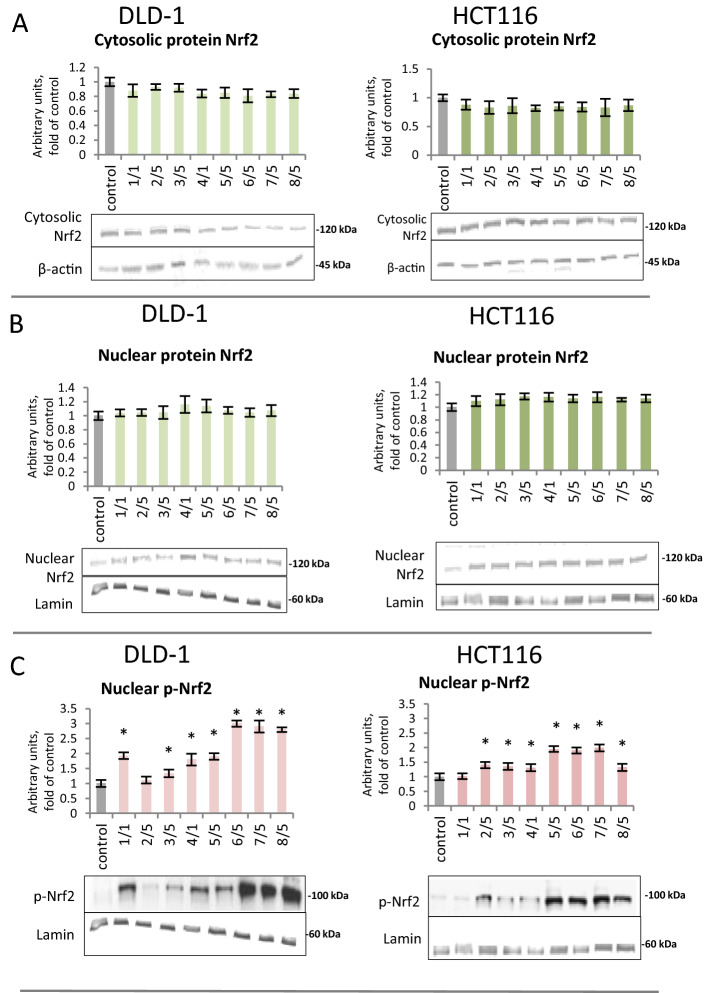

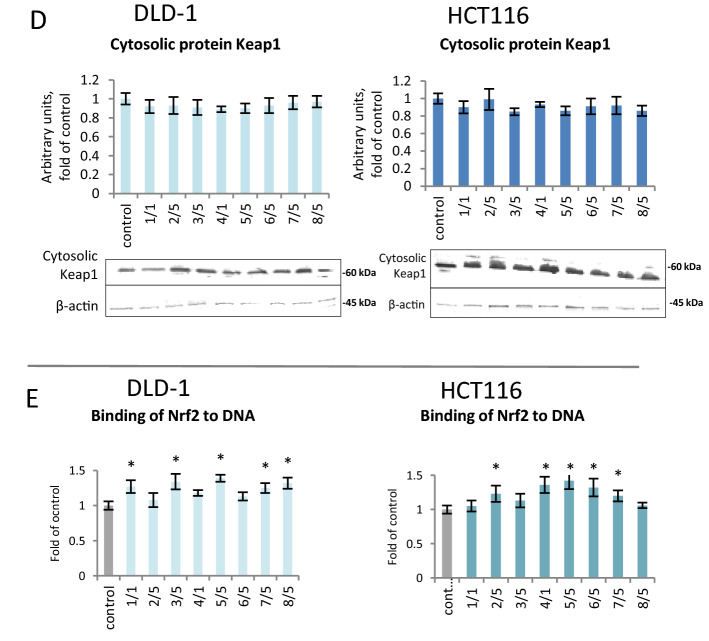
The effect of synthetic thioderivative chalcones compounds on Nrf2 activation in DLD-1 and HCT116 cells. (**A)** The level of Nrf2 protein in the cytosolic fraction. (**B) **The level of Nrf2 protein in the nuclear fraction. (**C) **The level of p-Nrf2 protein in the nuclear fraction. (**D) **The level of Keap1 protein in the cytosolic fraction. Representative Western immunoblots are presented under the graphs (**A–D**). (**E)** The level of Nrf2 binding to DNA. The values (mean ± SEM) were calculated as a relative change in the transcript or protein level in comparison with control cells (where control expression equals 1). The values from three separate experiments run in triplicate are presented. Asterisk: mean values were significantly different from the control group (p ≤ 0.05). Original scans of blots are shown in Supplementary Fig. S5.

To determine the influence of the tested compounds on the translocation of Nrf2 from the cytosol into the nucleus, the level of the Nrf2 protein in the cytosolic and nuclear fractions was assessed using Western blotting (Fig. 6A,B). Despite the lack of a statistically significant effect of the chalcones on the level of Nrf2 in the cytosol and nuclear fraction, we observed an increase in the nuclear level of p-Nrf2 in both tested cell lines. The strongest increase in the level of p-Nrf2 (180–200%) in the DLD-1 line was observed after treatment with chalcones **6, 7,** and **8** (Fig. 6C). In contrast, in the HCT116 line, the increase in p-Nrf2 was most noticeable in cells treated with derivatives **5, 6,** and **7**, which increased by 85%, 52%, and 80%, respectively.

All chalcones increased the binding of Nrf2 to a specific nucleotide sequence using ELISA. A statistically significant increase in Nrf2 binding was found after treatment with compounds **1, 3, 5, 7**, and **8** in DLD-1 cells and **2, 4, 5, 6**, and **7** in HCT116 cells (Fig. 6E).

**Figure 7:**
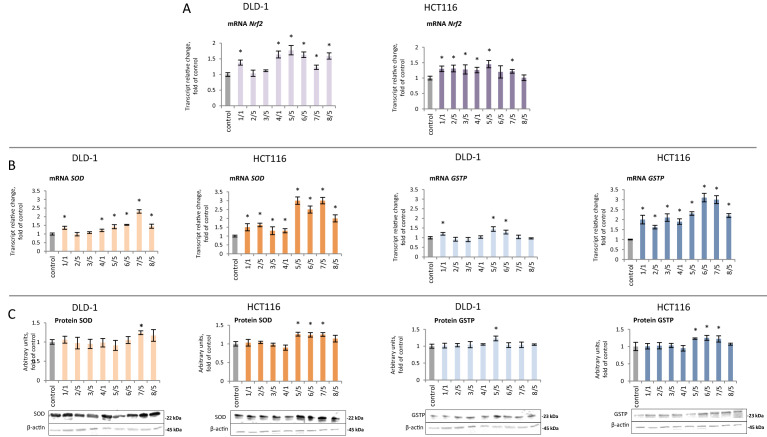
The effect of synthetic thioderivative chalcones compounds on Nrf2 expression in DLD-1 and HCT116 cells. (**A)** The level of *Nrf2* mRNA. (**B)** The level of mRNA of selected target genes of Nrf2: *SOD* and *GSTP*. (**C) **The levels of SOD and GSTP proteins in the cytosolic fraction. Representative Western immunoblots are presented under the graphs (**A–C**). The values (mean ± SEM) were calculated as a relative change in the transcript or protein level in comparison with control cells (where control expression equals 1). The values from three separate experiments run in triplicate are presented. Asterisk: mean values were significantly different from the control group (p ≤ 0.05). Original scans of blots are shown in Supplementary Fig. S6.

Nrf2 activation results in the expression of target genes. Quantitative analysis of *Nrf2* expression showed a significant increase in transcription in DLD-1 and HCT116 cells for the majority of synthetic chalcones. The highest increase in *Nrf2* transcription was observed upon treatment with derivative **5**, which caused an increase of 77% in the DLD-1 line and 45% in the HCT116 line (Fig. 7A). In order to explain—in part, at least—the mechanism of Nrf2 activation, the effect of tested chalcones on the Keap1 protein level was assessed (Fig. 6D). Protein Keap1 expression did not significantly affect as a result of treatment with all studied chalcones.

Activation of Nrf2 also resulted in the expression of antioxidant and detoxifying genes. All tested chalcones, except chalcones **2** and **3**, increased *SOD* transcription (from 21 to 130%) in DLD-1 cells (Fig. 7B). In HCT116 cells, increased levels of SOD mRNA and protein were observed under the influence of compounds **5, 6,** and **7**, but levels of *SOD* mRNA were significantly higher (Fig. 7B,C). The expression of GSTP (the gene that encodes the GSTP phase II enzyme) mRNA and protein were increased to the greatest extent (130–210%) by compounds **5, 6,** and **7** in the HCT116 line (Fig. 7B,C). However, only compound **5** significantly increased GSTP expression and protein levels in DLD-1 cells.

### The effect of thioderivative chalcones on the EGFR and Akt signaling

**Figure 8:**
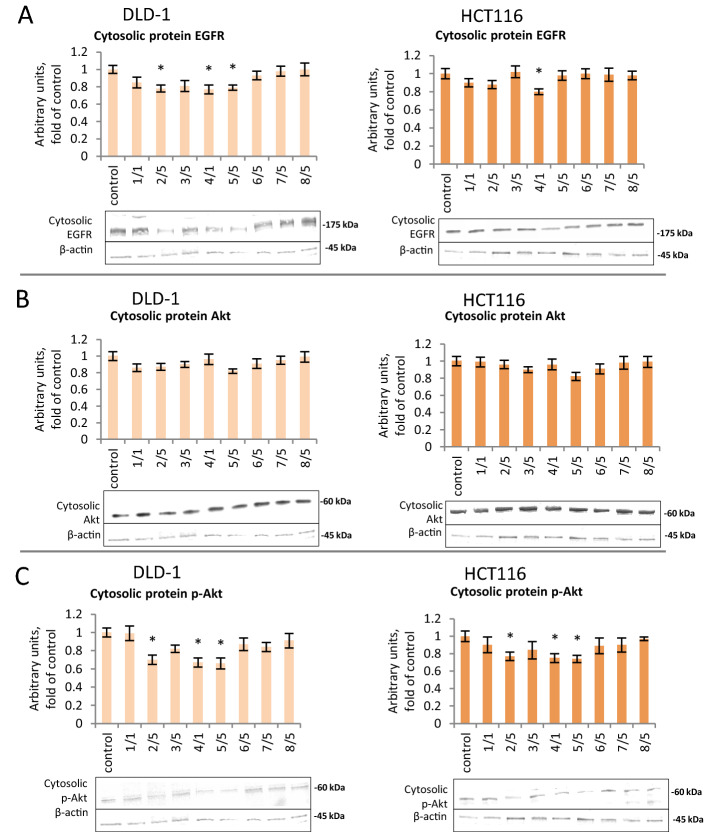
The effect of synthetic thioderivative chalcones compounds on EGFR activation in DLD-1 and HCT116 cells in the cytosolic fraction. (**A)** The level of EGFR protein. (**B) **The level of Akt protein. (**C) **The level of p-Akt protein. Representative Western immunoblots are presented under the graphs (**A–C**). The values (mean ± SEM) were calculated as a relative change in the protein level in comparison with control cells (where control expression equals 1). The values from three separate experiments run in triplicate are presented. Asterisk: mean values were significantly different from the control group (p ≤ 0.05). Original scans of blots are shown in Supplementary Fig. S7.

To assess possible interferences of the tested chalcones with the other signaling pathways involved in cell survival, EGFR, Akt and p-Akt were estimated. As shown in Fig. 8, chalcones modulated the EGFR, kinases Akt, and p-Akt level in both cancer cells. Western blot analysis demonstrated that compound **4** significantly decreased the level of EGFR in HCT116 cells, while compounds **2, 4,** and **5** in DLD-1 cells (Fig. 8A). Akt signaling pathways are implicated in cancer invasion. Thus, we then examined whether the invasion-suppressing activity of chalcones is related to the attenuation of expression of the protein Akt using western blot analysis. As shown in Fig. 8C compounds **2, 4** and **5** significantly reduced phosphorylation of p-Akt (Ser 473) in both tested cell lines.

### The effect of the most effective thioderivative chalcones on the cell cycle, apoptosis, and migration

**Figure 9:**
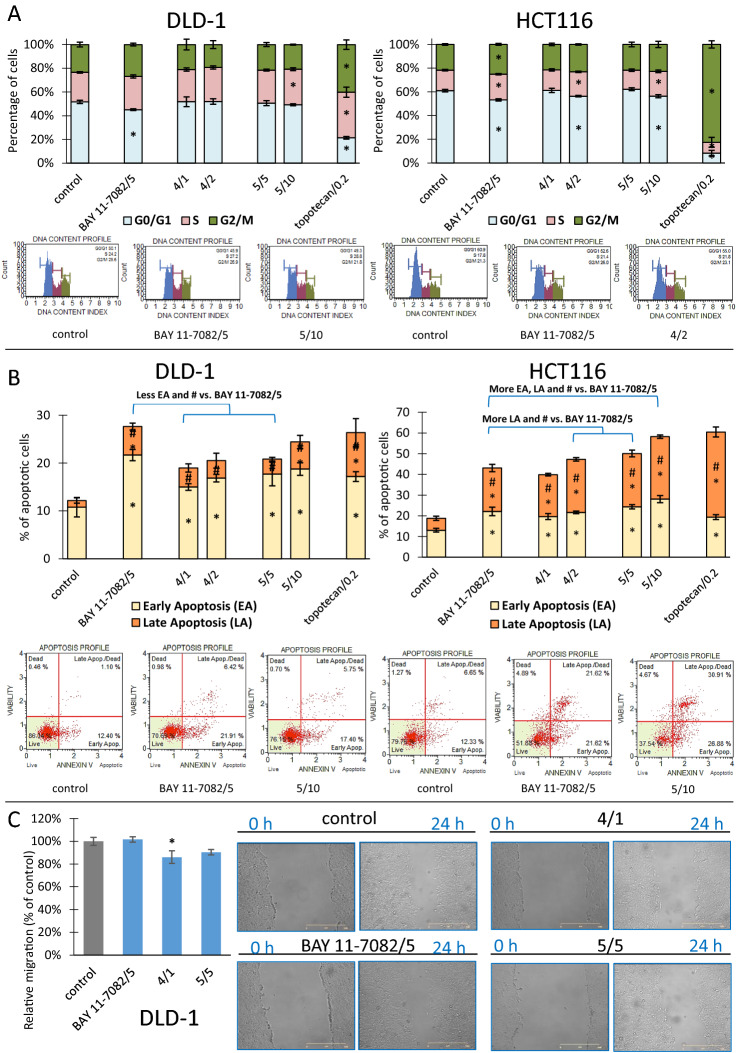
The impact of BAY 11-7082 (5 µM), chalcone **4** and chalcone **5**: (**A**) on cell cycle distribution in DLD-1 cells and HCT116 cells, (**B**) on induction of apoptosis in DLD-1 cells and HCT116 cells, (**C**) on migration of DLD-1 cells. (**A**) The percentage of cells in G1/G0, S and G2/M phases was analyzed by the flow cytometry after staining with propidium iodide (PI). Topotecan (0.2 µM) was used as a reference for cell cycle arrest. Exemplary plots are presented under the diagrams. Results (mean ± SEM) were calculated from three separate experiments. Asterisk: mean values were significantly different from the control group (p ≤ 0.05). (**B**) The percentage of cells in the early and late stage of the apoptosis was evaluated by the flow cytometry measurements after staining with Annexin V and 7-Aminoactinomycin D (7-AAD). Topotecan (0.2 µM) was used as a reference for induction of apoptosis. Exemplary plots are presented under the diagrams. Results (mean ± SEM) were calculated from three separate experiments. Asterisk: mean values were significantly different from the control group (or BAY 11-7082 treatment) and early apoptosis (EA) or late apoptosis (LA); Asterisk: mean values were significantly different from the control group (or BAY 11-7082 treatment) and Total apoptosis (p ≤ 0.05). **(C)** Wound healing assay was performed. Exemplary microscopic images of the scratch, which were taken at the beginning and after 24 h of incubation with the compounds, are shown. Results (mean ± SEM) were calculated from three separate experiments. Scale bar represents 500 μm. Asterisk: mean values were significantly different from the control group (p ≤ 0.05).

The most active compounds **4** and **5** were subjected to flow cytometric analysis of cell cycle distribution and apoptosis induction in standard and doubled concentrations (Fig. 9). Effects of BAY 11-7082 (5 µM) were also assessed to compare the potential of action of chalcones, and topotecan (0.2 µM) was used as a positive control. In DLD-1 cells, a decrease of G1/G0 cell cycle phase was shown for BAY 11-7082 and the action of chalcones was limited to a slightly enriching S phase in cells treated with **5** chalcone in higher concentration. For HCT116 cell line NF-κB inhibitor modulated the distribution of all cell cycle phases (decreased G1/G0 phases; increased S and G2/M phases). Similar results concerning G1/G0 and S phases were observed for cells exposed to chalcones **4** and **5** in higher concentrations (Fig. 9A).

In both cell lines, BAY 11-7082, **4** and **5** were able to induce apoptosis (Fig. 9B). For DLD-1 cell line, mainly early and total apoptotic cells populations were enriched. More total apoptotic cells were present after exposure to BAY 11-7082 comparing to chalcones, except comparable effect for **5** chalcone in higher concentration. In turn, for HCT116 cell line, an increase of early, late and total apoptosis was shown for all compounds. Moreover, chalcones **4** at 2 μM, and **5** at 5 μM and 10 μM induced a higher percentage of late and total apoptosis than BAY 11-7082.

For DLD-1 cell line, we were able to perform a wound healing assay to analyze the influence of the most active chalcones at its standard concentrations on cell migration (Fig. 9C). Wound healing after treatment with BAY 11-7082 (5 µM) was at the same level as DMSO-treated cells. For chalcones, we observed a tendency to decrease cell migration rate, with approximately 15% reduction for chalcone **4**.

### Molecular modeling of the most effective thioderivative chalcones

To determine whether the new thioderivative chalcones interact with the selected targets, molecular docking was performed to the structures of EGFR, Akt, IKKα/β and Keap1 found in the PDB database with their various inhibitors. The results were evaluated concerning the similarity of reproducing the interactions of the native inhibitors with the binding site of the target. Overall, all derivatives were docked in the active sites of the selected targets, but not all of them reproduced the binding mode of the native inhibitors. For illustration, compound **5** was used, which showed one of the most coherent docking results to high potent inhibitors (Supplementary Fig. 8). The almost perfect accommodation of compound **5** to the binding site of highly active inhibitors and the reproduction of key interaction sites suggest an important role for compound 5 in the signaling mechanisms studied.

Predicted ligand-based ADME/Tox properties of **1–8** showed (Supplementary Table 1) that all compounds exhibit very good ADME/Tox properties, only slight deviations from the preferred ranges are shown by compounds **5** and **6** for solubility and **1**, **4**, and **8** for skin permeability.

## Discussion

A recent study indicated that approaches such as surgical intervention, chemotherapy, and targeted therapy may be applied to treat colorectal cancer. Targeted therapies work by directly inhibiting cell proliferation, differentiation, and migration of cancerous cells. The tumor microenvironment, including local blood vessels and immune cells, might also be altered by targeted drugs in ways that impede tumor growth and enact stronger immune surveillance and response^17^.

Various pathways, such as the Wnt/β-catenin, NF-κB, EGFR, STAT3, and Nrf2-ARE pathways, mediate the initiation, progression, and migration of CRC and are thus ideal sites for targeted therapy. The interconnection of signaling pathways and aberrations in their activation mechanisms make it possible to use them as a target in cancer cells for potential therapeutic compounds. This approach is part of a new trend—*anakoinosis*. Inducing signal transduction in tumor cells modulates gene expression to alter communication between tumor tissues or between tumor tissue and the host. This concept is an alternative to conventional chemotherapy, which is usually either based on a single target or focused on a single area of the tumor. Unlike standard therapies, treatment protocols based on *anakoinosis* are less toxic and less likely to lead to drug resistance^18^.

Our previous study indicated that xanthohumol, a prenylated chalcone, significantly increased activation of the Nrf2 signaling pathway in HepG2 hepatoma cells and PANC-1 pancreatic cancer cells^19,20^. Additionally, we demonstrated that xanthohumol decreased the activation of NF-κB, the binding of both NF-κB subunits to DNA, and the expression of COX-2 in PANC-1 pancreatic cancer cells^20^.

The MTT assay is a common method for evaluating the cytotoxic effect of new substances on cancer cells. Thus, the MTT assay was used to estimate the biological activity of new synthetic compounds. Our findings revealed that the new derivative chalcones **1, 3, 4**, and **8**, to the most extent, inhibited DLD-1 cancer cell viability without affecting HCT116 cancer cells. DLD-1 and HCT116 cell lines derived separately from colorectal carcinomas have different chromosomal changes, which could explain the discrepancy^21^. Additionally, the normal HaCaT cells were less sensitive to the studied compounds than cancer cells DLD-1.

Signaling pathways such as Nrf2, NF-κB and STAT3 contribute to inflammation, and their cooperation is essential to carcinogenesis^22^. The NF-κB signaling pathway plays a key role in mediating inflammatory signals and controlling proinflammatory mediator production. Chalcone **4** downregulated NF-κB activation and the translocation of both subunits into the nuclear fraction, the binding of NF-κB to DNA and mRNA, and the transcriptional activation of COX-2 in both tested cell lines. This observation partially aligns with the findings on other chalcones, such as chalcones **5**–**8,** in both tested cell lines. The NF-κBp50 subunit is responsible for the binding of NF-κB to DNA and acts as a regulatory subunit of the NF-κB complex. The NF-κBp65 subunit is responsible for the initiation of transcription^23^. A recent study also indicated that NF-κBp50 can regulate apoptosis independently of the NF-κB complex^24^. Thus, the studied chalcones’ inhibitory effect on both subunits of NF-κB may be a pharmacologically potent CRC treatment. Moreover, inhibition of NF-κB and COX-2 expression by new chalcone derivatives might be essential for suppressing the aggressiveness of colorectal cancer.

STAT3 is closely associated with NF-κB signaling. Studies conducted in cancer cells showed that direct interactions between STAT3 and NF-κB contribute to inflammation^25^. Moreover, activation of these transcription factors promotes CRC cell proliferation and survival^22^.

Therefore, we examined whether chalcone derivatives influence the activation of the STAT3 pathway in this study. Chalcone **4** decreased the translocation of STAT3 from the cytosol to the nucleus and STAT3 phosphorylation and DNA binding, as well as downregulated mRNA expression in both the tested cell lines. A similar effect was observed for chalcone **2** in DLD-1 cells.

Nrf2 and NF-κB are directly involved in multiple carcinogenic processes through cooperation with multiple other signaling molecules, and Nrf2 and NF-κB signaling may affect cell differentiation and proliferation^26^. In our current study, we observed that chalcones **4** and **5** altered the rate of Nrf2 translocation to the nucleus, the binding of Nrf2 to DNA and the expression of Nrf2 in both tested cell lines. The binding of active Nrf2 to the antioxidant response element is thought to result in the induction of cytoprotective proteins, restoring redox balance and reducing inflammation^27^. Our results confirmed this mechanism. The increased expression and activation of Nrf2 were induced by chalcones **1, 4, 5, 6, 7,** and **8** in both tested cell lines. Because of the activation of Nrf2, the expression of its target genes, GSTP and SOD, was also increased. The proteins encoded by these genes play a crucial role in protection against reactive electrophiles produced as a result of xenobiotic exposure, as well as ROS, which are involved in colorectal carcinogenesis^28^.

The EGFR signaling pathway is a complex and tightly regulated process that is involved in the growth, proliferation, and survival of normal cells. Alterations within the EGFR signaling cascade, such as gene mutations, gene amplification, and protein overexpression, have been shown to contribute to colorectal carcinogenesis^29^. Therefore, the observed inhibition of this pathway, in combination with the phosphorylation of Akt on the critical residues Ser473, indicates the antitumor potential of compounds **4** and **5**.

Summarizing our research, we can indicate two compounds: **4** and **5,** as the most powerful chalcones for further study. Their protective effect appears to be associated with the inhibition of proinflammatory signaling mediated by NF-κB, EGFR and STAT3 and the activation of Nrf2. These findings provide a new mechanistic basis for the therapeutic application of new chalcone derivatives for the treatment of CRC.

## Methods

### General methods for chemistry

The methods used are very similar to the ones described in our previous publication^15^. All reagents and solvents were purchased from Sigma-Aldrich (including acetophenone **Ac03—**3,4,5-trimethoxyacetophenone, **Ald3—**3,4,5-trimethoxybenzaldehyde and **Ald11—**4-methylthiobenzaldehyde), Acros, Fluka, and POCH and were used as received. Reactions that involved air or moisture-sensitive reagents were performed in oven-dried glassware under an inert atmosphere of dry nitrogen with dried solvents, unless otherwise stated. The progress of all reactions was monitored on Merck precoated silica gel plates (with fluorescence indicator UV254) and visualized in UV light (*λ*_max_ 254 or 365 nm). Melting points were determined in capillary tubes on a Stuart SMP10 micro melting point apparatus and are uncorrected. ^1^H NMR and ^13^C NMR spectra were recorded at the Institute of Bioorganic Chemistry, Polish Academy of Sciences in Poznań, using Bruker 400 (400 MHz for ^1^H and 101 MHz for ^13^C), Bruker 500 (500 MHz for ^1^H and 126 MHz for ^13^C), and Bruker 700 (700 MHz for ^1^H and 176 MHz for ^13^C) spectrometers with TMS as an internal standard in CDCl_*3*_. Chemical shifts (*δ*) are quoted in parts per million (ppm) and are referred to as a solvent residual peak (CDCl_3_, *δ* 7.26 ppm for ^1^H and *δ* 77.0 ppm for ^13^C NMR). Coupling *δ* constants (*J*) are quoted in Hertz (Hz) and peaks are listed as singlet (s), doublet (d), triplet (t), multiplet (m) and doublet of doublets (dd). Dry flash column chromatography was carried out on Merck silica gel 60, particle size 40–63 μm or 15–40 μm using EZSafe low-pressure columns. All final target compounds were characterized and determined to be at least > 95% pure using a Waters ACQUITY UPLC H-class system equipped with a UV DAD and TQD Waters MS detector with electrospray ionization at the Department of Medicinal Chemistry, Institute of Pharmacology, Polish Academy of Sciences in Kraków. LC analysis was performed using a ACQUITY UPLC BEH C18 column (2.1 × 50 mm, 1,7 μm) at a flow rate of 0.3 mL/min (20–100% aqueous CH_3_CN over 3 min, 100% CH_3_CN over 0.5 min, and 100–20% aqueous CH_3_CN over 2.5 min).

### General procedure for synthesis of acetophenones (cf. schemes in supplementary materials)

Thiomethylbenzaldehydes (Ald4—3-bromo-4,5-dimethoxybenzaldehyde, Ald5—3-bromo-5-methoxy-4-methylthiobenzaldehyde, Ald6—3,5-dimethoxy-4-methylthiobenzaldehyde), (Ald01—3-methoxy-4-methylthiobenzaldehyde) and (Ald02—4-methoxy-3-methylthiobenzaldehyde) were obtained as described in Stefański et al. 2018^14^.

### Synthesis of alcoholes (Alk04Me–Alk06Me)

To a solution of benzaldehydes Ald4-6 (21 mmol) in anhydrous diethyl ether (25 ml) cooled to 0 °C, MeMgBr (10.5 ml, 31.5 mmol, 3.0 M soln in diethyl ether) was added dropwise and stirred under N_2_. The reaction mixture was allowed to warm to room temperature and monitored for completion by TLC. After 20 h, the reaction mixture was quenched with water (100 ml) acidified with 5% aqHCl to pH 5–6 and extracted with ethyl acetate (4 × 50 ml). The organic layer was separated, washed with brine, dried over anhydrous MgSO_4_, and concentrated in vacuo. The obtained yellowish oil was purified by flash column chromatography on silica gel (EtOAc/*n*-hexane, 4:10) to afford Alk04Me–Alk05Meas pale-yellow oils**.** The Alk06Meafter purification by flash column chromatography was subsequently recrystallized from EtOAc-petroleum ether to give product as pale-yellow crystals.

### Synthesis of acetophenones (Ac04–Ac06)

To a suspension of PCC (14 mmol, 3.02 g) in dichloromethane 80 ml, at 0 °C, a solution of appropriate alcohols (Alk04Me–Alk06Me) (10 mmol) in 20 ml of dichloromethane was added dropwise under N_2_ with vigorous stirring kept the reaction temperature about 0 °C. After the raction was completed (1 h), water was added. The reaction mixture was extracted with dichloromethane (3 × 50 ml), washed with brine, dried over anhydrous MgSO_4_, and concentrated in vacuo. Purification of crude products was performed by flash column chromatography on silica gel: Ac04—(EtOAc/n-hexane, 4:10), Ac05—(EtOAc/n-hexane, 3:1) subsequently recrystallized from EtOAc-petroleum ether, Ac06—(CH_2_Cl_2_) subsequently recrystallized from EtOAc-petroleum ether.

### General procedure for synthesis of thiomethylchalcones (Ald03–Ald02 1, Ald04–Ald01 2, Ac04–Ald02 3, Ac04–Ald11 4, Ac05–Ald01 5, Ac05–Ald02 6, Ac05–Ald11 7, Ac06–Ald02 8)

In the appropriate basic condition: 10 ml of methanol, ethanol and 0.1 ml 50% w/v aqueous solution of sodium or potassium hydroxide or 10% potassium hydroxide in methanol aldehydes (1 mmol) and acetophenones (1 mmol) was stirred overnight at room temperature. Then reaction mixture was acidified to pH 1–2 with 5% aqHCl and poured into crushed ice. The obtained crude products were recrystallized to afford the pure chalcones.

### Cell culture and viability assay

The DLD-1 and HCT116 colorectal carcinoma cell lines were purchased from ECACC. The HaCaT cell line of spontaneously immortalized keratinocytes was purchased from Cell Lines Service (Germany). The cells were cultured in Dulbecco’s Modified Eagle’s Medium (DMEM, Sigma-Aldrich, USA) with the addition of 10% FBS (EURx, Poland) and 1% antibiotic solution (penicillin and streptomycin) (Sigma-Aldrich, USA) at 37 ºC in an atmosphere with 95% humidity and 5% CO_2_.

The effect of the tested compounds on the viability of DLD-1, HCT116 and HaCaT cells was assessed by the MTT assay according to the standard protocol^19^.

### Western blot analysis

Cytosolic and nuclear extracts were prepared using a Nuclear/Cytosol Fractionation Kit (BioVision Research, USA). Cytosolic extracts (for NF-κBp65, NF-κBp50, COX-2, iNOS, STAT3, c-Myc, Bcl-xl, Nrf2, SOD, GSTP, EGFR, Akt, p-Akt, Keap1, IKKα/β and β-actin) or nuclear extracts (for NF-κBp65, NF-κBp50, STAT3, p-STAT3, Nrf2, p-Nrf2 and lamin) were separated on 7.5%, 10% or 12% SDS–PAGE slab gels. Then, the proteins were transferred to a nitrocellulose Immobilon-P membrane. After blocking for 2 h with 10% skimmed milk, proteins were incubated with primary antibodies against NF-κBp65, NF-κBp50, COX-2, iNOS, STAT3, p-STAT3, c-Myc, Bcl-xl, Nrf2, p-Nrf2, SOD, GSTP, EGFR, Akt, p-Akt, Keap1, IKKα/β, β-actin, and lamin. Alkaline phosphatase (AP)-labeled anti-rabbit IgG, anti-goat IgG, and anti-mouse IgG secondary antibodies (Bio-Rad Laboratories, USA), as well as horseradish peroxidase (HRP)-conjugated anti-mouse IgG (Boster Bio, USA) secondary antibodies were used in the staining reaction. Bands were visualized by the AP Conjugate Substrate Kit NBT/BCIP or the chemiluminescent HRP substrate of the Clarity ECL Kit (Bio–Rad Laboratories, USA). The amount of immunoreactive product in each lane was determined using a ChemiDoc Imaging System (Bio–Rad Laboratories, USA). Values were calculated as relative absorbance units (RQ) per mg of protein and expressed as a percentage of the control.

### Elisa analysis

To estimate NF-ΚB (p50 and p65 subunits) STAT3 and Nrf2 activation, enzymatic immunoassays with the Transcription Factor ELISA Kit (TransAM™NF-κB, TransAM™STAT3/TransAM™Nrf2, Active Motif, Carlsbad CA, USA) were used following the manufacturer’s protocol. Briefly, proper consensus-site double-strand oligonucleotides (NF-κB—5′-GGGACTTTCC-3′; STAT3-5’—TTCCCGGAA-3’; Nrf2-5ʹ—GTCACAGTGACTCAGCAGAATCTG-3ʹ) were immobilized on an ELISA plate and incubated for one hour with the nuclear extracts. The activated subunits bound to DNA were recognized with a specific primary antibody and detected with an HRP-conjugated secondary antibody. The number of subunits was determined by spectrophotometry at λ = 450 nm. The number of p50, and p65 subunits, STAT3, as well as the amount of Nrf2 contained in the DNA-binding complex, corresponded to the activated NF-κB, STAT3, or Nrf2 transcription factors.

### Quantitative real-time PCR (qRT–PCR)

Total RNA from cells was isolated with the GeneMatrix Universal DNA/RNA/Protein Purification Kit (EurX, Poland). For the qRT-PCR analyses, the Maxima SYBR Green I/ROX qPCR Master Mix (2×) (Thermo Fisher Scientific, USA) and the thermal cycler LightCycler (Roche, Germany) were used. The protocol started with 5 min of enzyme activation at 95 °C, followed by 40–50 cycles at 95 °C for 15 s, 56 °C for 20 s, 72 °C for 40 s, and final elongation at 72 °C for 5 min. Melting curve analysis was used for amplicon verification. The expression levels of *TATA-box-binding protein* (*TBP*) and *porphobilinogen deaminase* (*PBGD*) were used to normalize the data. The Pfaffl comparative method was used to calculate fold change. Primers were designed using Beacon Designer software (the sequences of primers used to analyze genes are listed in Table 2 of the supplementary materials).

### Cell cycle distribution

The analysis of the cell cycle distribution was performed by propidium iodide (PI) staining. The addition of RNase A improved the specificity of PI binding to DNA. The Muse Cell Cycle Kit (Luminex, USA) and flow cytometric detection were performed with the Muse Cell Analyzer (Merck, Germany), according to the manufacturer’s protocol. Briefly, cells were seeded (2 × 10^5^ cells per well) in 6-well plates and on the following day the analyzed compounds were added. Topotecan (Sigma-Aldrich, USA) was used as a positive control of cell cycle arrest. After 48 h cells were collected by trypsinization, fixed in cold 70% ethanol and stored at –20 °C. Then, fixed cells were collected and stained with PI, subjected to 30 min incubation at room temperature in the dark and analyzed by flow cytometry. Three independent experiments were performed. Data were analyzed by Muse 1.5 Analysis Software (Merck, Germany).

### Apoptosis

Apoptosis was assessed by the detection of phosphatidylserine externalization based on Annexin V staining combined with fluorescent DNA intercalator 7-Aminoactinomycin D (7-AAD) what allowed for the discrimination between early and late apoptotic cells. The analysis was performed by flow cytometry using the Muse Annexin V & Dead Cell Kit (Luminex, USA) and the Muse Cell Analyzer (Merck, Germany), according to the manufacturer’s protocol. Cells were seeded (2 × 10^5^ cells per well) in six-well plates and on the following day the analyzed compounds were added. Topotecan (Sigma-Aldrich, USA) was used as a positive control of apoptosis induction. After 48 h cells were collected by trypsinization, stained and after 20 min incubation at room temperature in the dark analyzed by flow cytometry. Three independent experiments were performed. Data were analyzed by Muse 1.5 Analysis Software (Merck, Germany).

### Cell migration

Cell migration was assessed by the wound healing assay. DLD-1 cells were seeded into 24-well plates (5 × 10^5^ cells per well) and were grown overnight to reach confluency. A scratch was performed using a 10 μl tip, wells were rinsed twice with PBS buffer to remove detached cells, and fresh medium with reduced FBS concentration (0.5%) containing the tested compounds was added. Subsequently, representative microscopic photographs of scratch areas were taken by JuLI FL microscope (NanoEntek, South Korea) direct after the addition of compounds and after 24 h. Area covered by cells (%) was assessed using JuLI FL software and the difference in the coverage of the growth area by DLD-1 cells between 0 and 24 h time points was calculated for each well. The experiment was repeated three times. The relative effect of the tested compounds on cell migration was calculated by comparing the difference in cell coverage area between compound-treated cells and DMSO-vehicle control (tested compound [area 24 h − area 0 h] / DMSO-vehicle control [area 24 h − area 0 h]).

### Molecular modeling

The molecular docking of the synthesized library **1–8** was performed using the Glide algorithm from Schrödinger Suite. The 3D conformations of the ligands were generated using LigPrep, and the appropriate ionization states at pH  7.4 ± 0.5 were assigned using Epik v5.3.

The Protein Data Bank was screened to select the high-resolution crystal structures for the targets: EGFR (PDB ID: 4I23, 5UGB, 6VH4), Akt (PDB ID: 3QKL, 3QKM, 4EKL, 4GV1), IKKα/β (PDB ID: 3RZF), and Keap1 (PDB ID: 4XMB, 6QMC, 6QME, 6QNK, 6zF1). Next, the Protein Preparation Wizard was used to assign the bond orders and appropriate amino acid ionization states and to check for steric clashes. The receptor grids were generated (OPLS3e force field) by centering the grid box with a size of 12 Å on the inhibitor structure. Automated flexible docking was performed at the standard precision (SP) level with the generation of ten poses per ligand.

### ADME/Tox prediction

The ligand-based ADME/Tox prediction was performed for **1–8** using the QikProp algorithm from Schrödinger Suit. Out of 52 calculated descriptors and properties, the following were selected to characterize the library of obtained compounds: QPlogPo/w (octanol/water partition coefficient; recommended values are between –2.0 and 6.5); QPlogS (aqueous solubility, recommended values are between –6.5 and 0.5); QPlogHERG (predicted IC50 value for blockage of HERG K^+^ channels, recommended values are below –5); QPPCaco (apparent Caco-2 cell permeability in nm/sec., recommended values > 500); QPlogBB (brain/blood partition coefficient, recommended values are between –3.0 and 1.2); QPPMDCK (apparent MDCK cell permeability in nm/sec.,) recommended values > 500); QPlogKp (skin permeability, log K_p_, recommended values are between –8.0 and –1.0); QPlogKhsa (of binding to human serum albumin, recommended values are between –1.5 and 1.5).

### Statistical analysis

Statistical analysis was performed using GraphPad InStat version 3.10 (GraphPad Software, USA), assuming the significance level of changes to be p < 0.05. Student’s *t*-test was used to assess the similarity of the experimental groups to the control. Additionally, Dunnett’s post hoc test was used to assess the significance of the differences between experimental groups to the group treated with BAY 11-7082 (Sigma-Aldrich, USA).

## Supplementary Information

Supplementary Information.

## Data Availability

The datasets generated during and/or analysed during the current study are available from the corresponding author on reasonable request.
