# Supplementary material for: The synthesis of novel thioderivative chalcones and their influence on NF-κB, STAT3 and NRF2 signaling pathways in colorectal cancer cells
Source: Sci Rep. 2022 Sep 1;12:14915. doi: 10.1038/s41598-022-18981-4 (PMC9436958; doi:10.1038/s41598-022-18981-4)
Supplement: Supplementary file 1 — Supplementary Information. [file 41598_2022_18981_MOESM1_ESM.pdf]

Supplementary Table 1. Predicted ADME/Tox descriptors or properties for the thioderivative chalcones 1–8.

Compound	QPlogPo/w	QPlogS	QPlogHERG	QPPCaco	QPlogBB	QPPMDCK	QPlogKp	QPlogKhsa
1	4.6	-5.2	-5.6	3719	-0.4	3391	-0.9	0.3
2	5.0	-5.8	-5.7	3716	-0.1	7947	-1.0	0.5
3	5.0	-5.8	-5.6	3714	-0.1	7824	-1.0	0.5
4	4.9	-5.5	-5.6	3713	-0.1	7967	-0.9	0.5
5	5.6	-6.6	-5.8	3726	-0.1	10000	-1.0	0.7
6	5.6	-6.7	-5.8	3719	-0.1	10000	-1.0	0.7
7	5.4	-6.4	-5.8	3719	0.0	10000	-1.0	0.7
8	5.2	-6.2	-5.9	3718	-0.3	5499	-0.9	0.5

Supplementary Table 2. Sequences of primers used in the qRT-PCR reaction (Laboratory of Sequencing and Synthesis of Oligonucleotides of the Institute of Biochemistry and Biophysics of the Polish Academy of Sciences, Poland).

Gene	Primier F (5’→3’)	Primer R (5’→3’)
NF-κB p50	**5’ATCATCCACCTTCATTCTCAA**	**5’AATCCTCCACCACATCTTCC**
NF-κB p65	**5’CGCCTGTCCTTTCTCATC**	**5’ACCTCAATGTCCTCTTTCTG**
COX-2	**5’CGCCTGTCCTTTCTCATC**	**5’CAGCCCGTTGGTGAAAGC**
iNOS	**5’AGGAGATGCTGAACTACG**	**5’GGATGGTGACTCTGACTC**
STAT3	**5’GCTTCTCCTTCTGGGTCTG**	**5’AGGCTTAGTGCTCAAGATGG**
c-MYC	**5’TTACAACACCCGAGCAAG**	**5’ AATCCAGCGTCTAAGCAG**
Bcl-xl	**5’AAGCGTAGACAAGGAGATGC**	**5’CAGCGGTTGAAGCGTTCC**
Nrf2	**5′ATTGCTACTAATCAGGCTCAG**	**5′GTTTGGCTTCTGGACTTGG**
SOD	**5’CGACAGAAGGAAAGTAATG**	**5’TGGATAGAGGATTAAAGTGAGG**
GSTP	**5’GCAAATACATCTCCCTCATC**	**5’AGGTTGTAGTCAGCGAAG**
**Reference genes**		
PBGD	**5’TCAGATAGCATACAAGAGACC**	**5’TGGAATGTTACGAGCAGTG**
TBP	**5’GGCACCACTCCACTGTATC**	**5’GGGATTATATTCGGCGTTTCG**

Schemes for synthesis routes:

Synthesis of acetophenone

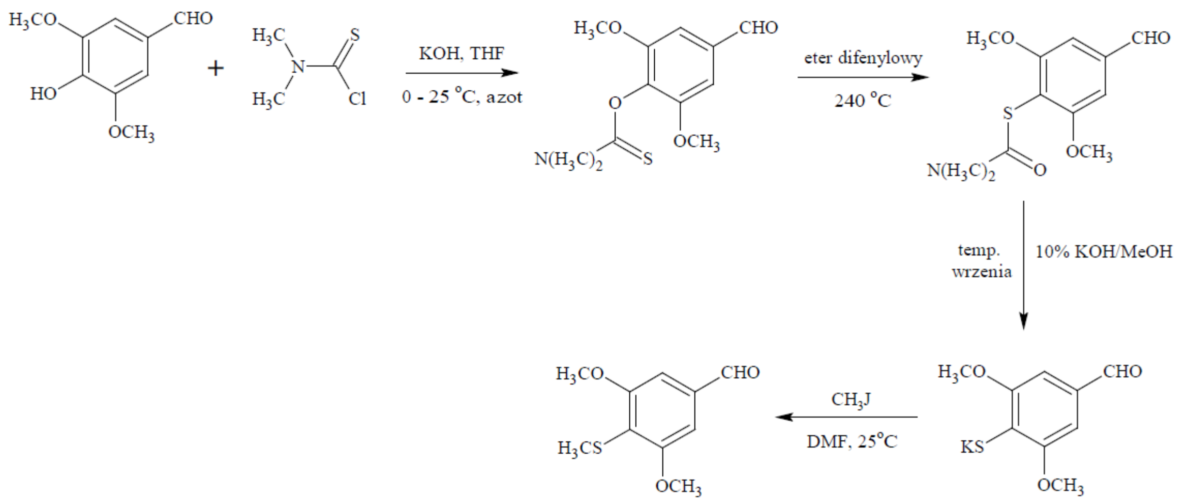

Synthesis of alcohols and acetophenones

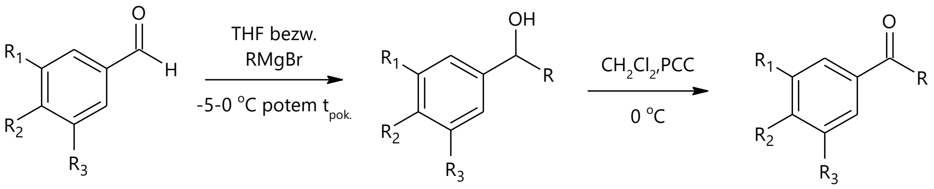

R=CH_3 - AlkXMe (d) **R=CH_3 - AcX (a)**

R=C_2H_5 - AlkXEt (e) **R=C_2H_5 - PrX (c)**

X = 01, 02, 0n...

Synthesis of thiomethyl chalcones

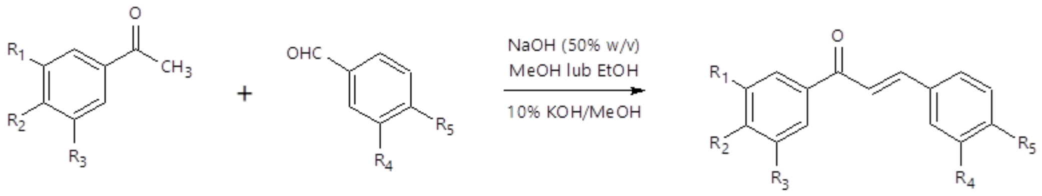

(a) **(b)**

chalkon

Ac-Ald

Spectral data of the synthesized compounds(Supplementary file)

1-(3-bromo-4,5-dimehtoxyphenyl)ethanol (Alk04Me)

Pale yellow oil. Yield 55% (3.016 g, 11.55 mmol).R_f (EtOAc/n-hexane, 1:1) 0.54. **^{1}H NMR** (400 MHz, $CDCl_3$) ppm (δ): 7.10 (d, J = 1.7 Hz, 1H, Ar), 6.88 (d, J = 1.9 Hz, 1H, Ar), 4.80 (q, J = 6.4 Hz, 1H, C**H**-OH), 3.86 (s, 3H, OCH_3), 3.83 (s, 3H, OCH_3), 1.46 (d, J = 6.4 Hz, 3H, CH_3).

^{13}C NMR (101 MHz, $CDCl_3$) ppm (δ): 153.66, 145.45, 143.06, 121.51, 117.42, 108.80, 69.66 (CH-OH), 60.51 (OCH_3), 56.04 (OCH_3), 25.17 (CH_3).

1-(3-bromo-5-methoxy-4-methythiophenyl)ethanol (Alk05Me)

Pale yellow oil. Yield 69% (4.016 g, 14.49 mmol).R_f (EtOAc/n-hexane, 1:1) 0.51. **^{1}H NMR** (400 MHz, $CDCl_3$) ppm (δ): 7.24 (d, J = 1.7 Hz, 1H,Ar), 6.90 (d, J = 1.6 Hz, 1H, Ar), 4.85 (q, J = 6.3 Hz, 1H, C**H**-OH), 3.92 (s, 3H, OCH_3), 2.39 (s, 3H, SCH_3), 1.49 (d, J = 6.5 Hz, 3H, CH_3).

^{13}C NMR (101 MHz, $CDCl_3$) ppm (δ): 161.04, 148.34, 130.61, 124.30, 122.23, 107.16, 69.65 (CH-OH), 56.34 (OCH_3), 25.23 (CH_3), 18.20 (SCH_3).

1-(3,5-dimethoxy-4-methythiophenyl)ethanol (Alk06Me)

Pale yellow crystals. Yield 64% (3.069 g, 13.44 mmol).mp 79-82 °C. R_f (EtOAc/n-hexane, 1:1) 0.40. **^{1}H NMR** (500 MHz, $CDCl_3$) ppm (δ): 6.60 (s, 2H), 4.88 (q, J = 6.4 Hz, 1H, Ar), 3.91 (s, 6H, 2xOCH_3), 2.35 (s, 3H, SCH_3), 1.91 (s, 1H), 1.50 (d, J = 6.5 Hz, 3H, CH_3).**^{13}C NMR** (126 MHz, $CDCl_3$) ppm (δ): 160.64, 147.77, 101.09, 70.59 (CH-OH), 56.19 (OCH_3), 25.23 (CH_3), 17.78 (SCH_3).

3-bromo-4,5-dimethoxyacetophenone(Ac04)

Pale yellow crystals. Yield 38% (0.985 g, 3.8 mmol).mp 66-68°C (lit. 63-65°C [28]). R_f (EtOAc/n-hexane, 4:10) 0.67. **^{1}H NMR** (400 MHz, $CDCl_3$) ppm (δ): 7.73 (d, J = 2.0 Hz, 1H, Ar), 7.48 (d, J = 2.0 Hz, 1H, Ar), 3.92 (s, 6H, OCH_3), 2.57 (s, 3H, OCH_3). **^{13}C NMR** (101 MHz, $CDCl_3$) ppm (δ): 195.86 (CH_3-**C**=O), 153.62 (Ar), 133.78, 128.78, 126.18, 117.31, 110.77, 60.74 (OCH3), 56.22 (OCH3), 26.38 (**C**H_3-C=O).

3-bromo-5-methoxy-4-methylthioacetophenone (Ac05)

Pale yellow crystals. Yield 29% (0.798 g, 2.9 mmol).mp59-62°C. R_f (EtOAc/n-hexane, 1:1) 0.77.

^{1}H NMR (400 MHz, $CDCl_3$) ppm (δ): 7.78 (d, J = 1.7 Hz, 1H, Ar), 7.42 (d, J = 1.7 Hz, 1H, Ar), 3.96 (s, 3H, OCH_3), 2.58 (s, 3H, SCH_3), 2.48 (s, 3H, CH_3). **^{13}C NMR** (101 MHz, $CDCl_3$)

ppm (δ): 196.11 (CH_3-**C**=O), 160.61, 137.66, 132.36, 129.15, 125.77, 108.52, 56.44 (OCH3), 26.53 (**C**H_3-C=O), 18.13 (SCH_3).

3,5-dimethoxy-4-methylthioacetophenone (Ac06)

Pale yellow crystals. Yield 33% (0.747 g, 3.3 mmol).mp82-86°C. R_f (EtOAc/n-hexane, 1:1) 0.65.

^{1}H NMR (400 MHz, $CDCl_3$) ppm (δ): 7.14 (s, 2H), 3.96 (s, 6H, 2xOCH_3), 2.61 (s, 3H, SCH_3), 2.44 (s, 3H, CH_3).**^{13}C NMR** (101 MHz, $CDCl_3$) ppm (δ): 197.22, 160.09, 137.23, 119.06, 103.81, 56.34 (OCH3), 26.59 (**C**H_3-C=O), 17.53 (SCH_3).

***1**:3-(4-methoxy-3-methylthiophenyl)-1-(3,4,5-trimethoxyphenyl)prop-2-en-1-one (Ac03-Ald02)*

Yellow crystals (EtOH). Yield 60% (0.225 g, 0.6 mmol) – basic condition 10%KOH/MeOH. mp 125-128 °C. R_f (EtOAc/n-hexane, 4:10) 0.35. HPLC: 99.3%, tR = 3.33 min.**^{1}H NMR** (500 MHz, $CDCl_3$) ppm (δ): 7.79 (d, *J* = 15.6 Hz, 1H, -CH=CH-), 7.49 (dd, *J* = 8.4, 2.0 Hz, 1H, C6'-H), 7.45 (d, *J* = 1.9 Hz, 1H, ArH), 7.37 (d, *J* = 15.6 Hz, 1H, -CH=CH-), 7.29 (s, 2H, ArH), 6.90 (d, *J* = 8.4 Hz, 1H, ArH), 3.97 (s, 9H, OCH3), 3.96 (s, 3H, OCH3), 2.51 (s, 3H, SCH_3). **^{13}C NMR** (126 MHz, $CDCl_3$) ppm (δ): 189.38, 158.36, 153.16, 144.45, 142.41, 133.78, 128.18, 128.07, 127.02, 126.36, 120.01, 110.17, 106.12, 61.00 (OCH_3), 56.46 (OCH_3), 56.09 (OCH_3), 14.85 (SCH_3). LRMS (ES+) 374.90 [M^+]

***2**:3-(3-methoxy-4-methylthiophenyl)-1-(3-bromo-4,5-dimethoxyphenyl)prop-2-en-1-one (Ac04-Ald01)*

Yellow crystals (iPrOH). Yield 43% (0.182 g, 0.43 mmol) – basic condition NaOH/MeOH. mp 137-139 °C. R_f (EtOAc/n-hexane, 4:10) 0.82. HPLC: 97.5%, tR = 3.78 min. **^{1}H NMR** (400 MHz, $CDCl_3$) ppm (δ): 7.83 – 7.74 (m, 2H, Ar), 7.56 (d, *J* = 2.0 Hz, 1H, Ar), 7.39 (d, *J* = 15.6 Hz, 1H, -CH=CH-), 7.28 (dd, *J* = 8.1, 1.8 Hz, 1H, Ar), 7.15 (d, *J* = 8.1 Hz, 1H, Ar), 7.06 (d, *J* = 1.6 Hz, 1H, Ar), 3.98 (s, 3H, OCH_3), 3.96 (s, 3H, OCH_3), 3.95 (s, 3H, OCH_3), 2.48 (s, 3H, SCH_3). **^{13}C NMR** (176 MHz, $CDCl_3$) ppm (δ): 187.93 (C=O), 155.97, 153.82, 150.38, 145.29, 135.01, 132.02, 132.01, 125.63, 124.82, 122.36, 119.98, 117.25, 111.47, 108.78, 60.79 (OCH_3), 56.29 (OCH_3), 56.00 (OCH_3), 14.22 (SCH_3). LRMS (ES+) 424.81 $[M+H]^+$

***3**: 3-(4-methoxy-3-methylthiophenyl)-1-(3-bromo-4,5-dimethoxyphenyl)prop-2-en-1-one (Ac04-Ald02)*

Yellow crystals (Diisopropyl ether). Yield 30% (0.127 g, 0.3 mmol) – basic condition NaOH/EtOH. mp 112-115 °C. R_f (EtOAc/n-hexane, 4:10) 0.58. HPLC: 97.6%, tR = 3.76 min. **^{1}H NMR** (400 MHz, $CDCl_3$) ppm (δ): 8.21 (d, *J* = 2.2 Hz, 1H, Ar), 7.84 (d, *J* = 1.9 Hz, 1H, Ar), 7.78 (d, *J* = 15.6 Hz, 1H, -CH=CH-), 7.68 (dd, *J* = 8.4, 2.2 Hz, 1H, Ar), 7.43 (d, *J* = 2.1 Hz, 1H, Ar), 7.33 (d, *J* = 15.6 Hz, 1H, -CH=CH-), 6.98 (d, *J* = 8.5 Hz, 1H, Ar), 3.96 (s, 3H,

OCH_3), 3.96 (s, 3H, OCH_3), 3.95 (s, 3H, OCH_3), 2.50 (s, 3H, SCH_3). **^{13}C NMR** (176 MHz, $CDCl_3$) ppm (δ): 187.66 (C=O), 156.48, 153.77, 145.01, 143.60, 134.76, 134.09, 127.19, 126.30, 125.81, 123.59, 120.34, 119.20, 111.40, 110.14, 60.78 (OCH_3)., 56.33 (OCH_3)., 56.13 (OCH_3)., 14.83 (SCH_3). LRMS (ES+) 424.75 $[M+H]^+$

***4**: 3-(4-methylthiophenyl)-1-(3-bromo-4,5-dimethoxyphenyl)prop-2-en-1-one (Ac04-Ald11)*

Pale yellow crystals (Diisopropyl ether). Yield 46% (0.181 g, 0.46 mmol) – basic condition 10%KOH/MeOH. mp 137-139 °C. R_f (EtOAc/n-hexane, 4:10) 0.67. HPLC: 98.5%, tR = 3.88 min. **^{1}H NMR** (700 MHz, $CDCl_3$) ppm (δ): 7.86 – 7.82 (m, 2H, Ar), 7.81 (d, *J* = 15.5 Hz, 1H, -CH=CH-), 7.60 (d, *J* = 8.3 Hz, 2H, Ar), 7.58 (d, *J* = 1.9 Hz, 1H, Ar), 7.45 (d, *J* = 15.5 Hz, 1H, -CH=CH-), 7.30 (s, 1H, Ar), 3.99 (s, 3H, OCH_3), 3.97 (s, 3H, OCH_3), 2.56 (s, 3H, SCH_3). **^{13}C NMR** (176 MHz, $CDCl_3$) δ 187.85 (C=O), 153.80, 150.39, 144.80, 142.74, 134.97, 131.13, 129.21, 128.92, 125.93, 125.66, 124.18, 119.94, 117.30, 111.42, 60.78 (OCH_3)., 56.27 (OCH_3)., 15.10 (SCH_3). LRMS (ES+) 394.69 $[M+H]^+$

***5**:3-(3-methoxy-4-methylthiophenyl)-1-(3-bromo-5-methoxy-4-methylthiophenyl)prop-2-en-1-one (Ac05-Ald0)*

Yellow crystals (MeOH). Yield 49% (0.215 g, 0.49 mmol) – basic condition NaOH/EtOH. mp 130-133 °C. R_f (EtOAc/n-hexane, 4:10) 0.53. HPLC: 98.7%, tR = 3.96 min. **^{1}H NMR** (400 MHz, $CDCl_3$) ppm (δ): 7.85 (d, *J* = 1.6 Hz, 1H, Ar), 7.80 (d, *J* = 15.6 Hz, 1H, -CH=CH-), 7.48 (d, *J* = 1.7 Hz, 1H, Ar), 7.37 (d, *J* = 15.5 Hz, 1H, -CH=CH-), 7.28 (dd, *J* = 8.0, 1.7 Hz, 1H, Ar), 7.15 (d, *J* = 8.1 Hz, 1H, Ar), 7.06 (d, *J* = 1.7 Hz, 1H, Ar), 4.00 (s, 3H, OCH_3), 3.98 (s, 3H, OCH_3), 2.50 (s, 3H, SCH_3), 2.48 (s, 3H, SCH_3). **^{13}C NMR** (126 MHz, $CDCl_3$) ppm (δ): 188.29 (C=O), 160.77, 155.98, 145.77, 139.11, 132.29, 131.90, 131.78, 129.12, 125.24, 124.80, 122.45, 120.00, 109.26, 108.81, 56.52 (OCH_3), 56.00 (OCH_3), 18.19 (SCH_3), 14.20 (SCH_3).LRMS (ES+) 440.67 $[M+H]^+$

***6**: 3-(4-methoxy-3-methylthiophenyl)-1-(3-bromo-5-methoxy-4-methylthiophenyl)prop-2-en-1-one (Ac05-Ald02)*

Yellow crystals (MeOH). Yield 57% (0.250 g, 0.57 mmol) – basic condition NaOH/EtOH. mp 131-133 °C. R_f (EtOAc/n-hexane, 4:10) 0.47. HPLC: 97.2%, tR = 3.94 min. **^{1}H NMR** (400 MHz, $CDCl_3$) ppm (δ): 7.84 (d, *J* = 1.7 Hz, 1H, Ar), 7.79 (d, *J* = 15.6 Hz, 1H, -CH=CH-), 7.48 (dd, *J* = 7.7, 1.9 Hz, 2H, Ar), 7.43 (d, *J* = 2.1 Hz, 1H, Ar), 7.31 (d, *J* = 15.6 Hz, 1H, -CH=CH-), 6.88 (d, *J* = 8.4 Hz, 1H, Ar), 4.00 (s, 3H, OCH_3), 3.96 (s, 3H, OCH_3), 2.52 (s, 3H, SCH_3), 2.50 (s, 3H, SCH_3). **^{13}C NMR** (126 MHz, $CDCl_3$) ppm (δ): 188.33 (C=O), 160.76, 158.58, 145.49, 139.22, 131.65, 129.17, 128.25, 127.87, 127.29, 126.38, 125.24, 119.23, 110.16, 109.26, 56.52 (OCH_3), 56.09 (OCH_3), 18.19 (SCH_3), 14.83 (SCH_3). LRMS (ES+) 440.81 $[M+H]^+$

***7**: 3-(4-methylthiophenyl)-1-(3-bromo-5-methoxy-4-methylthiophenyl)prop-2-en-1-one (Ac05-Ald11)*

Yellow crystals (iPrOH). Yield 64% (0.262 g, 0.64 mmol) – basic condition NaOH/EtOH. mp 142-144 °C. R_f (EtOAc/n-hexane, 4:10) 0.58. HPLC: 100%, tR = 4.05 min. **^{1}H NMR** (400 MHz, $CDCl_3$) ppm (δ): 7.84 (d, *J* = 1.7 Hz, 1H, Ar), 7.80 (d, *J* = 15.6 Hz, 1H, -CH=CH-), 7.58 (d, *J* = 8.2 Hz, 2H, Ar), 7.48 (d, *J* = 1.7 Hz, 1H, Ar), 7.40 (d, *J* = 15.6 Hz, 1H, -CH=CH-), 7.27 (d, *J* = 8.2 Hz, 2H, Ar), 3.99 (s, 3H, OCH_3), 2.53 (s, 3H, SCH_3), 2.50 (s, 3H, SCH_3).**^{13}C NMR** (101 MHz, $CDCl_3$) δ 188.13 (C=O) , 160.71, 145.21, 142.94, 139.02, 131.74, 130.98, 129.22, 129.12, 128.94, 125.88, 125.22, 124.15, 119.90, 109.17, 56.46 (OCH_3), 18.14 (SCH_3), 15.03 (SCH_3).LRMS (ES+) 410.89 $[M+H]^+$

***8**:3-(4-methoxy-3-methylthiophenyl)-1-(3,5-dimethoxy-4-methylthiophenyl)prop-2-en-1-one (Ac06-Ald02**8**)*

Yellow crystals (EtOH). Yield 55% (0.215 g, 0.55 mmol) – basic condition NaOH/MeOH. mp 161-163 °C. R_f (EtOAc/n-hexane, 1:1) 0.71. HPLC: 98.6%, tR = 3.54 min. **^{1}H NMR** (500 MHz, $CDCl_3$) ppm (δ): 7.78 (d, *J* = 15.6 Hz, 1H, -CH=CH-), 7.47 (dd, *J* = 8.4, 2.0 Hz, 1H, ArH), 7.43 (d, *J* = 2.0 Hz, 1H, ArH), 7.33 (d, *J* = 15.6 Hz, 1H, -CH=CH-), 7.17 (s, 2H, ArH), 6.88 (d, *J* = 8.4 Hz, 1H, ArH), 3.99 (s, 6H, OCH_3), 3.96 (s, 3H, OCH_3), 2.49 (s, 3H, SCH_3), 2.45 (s, 3H, SCH_3). **^{13}C NMR** (126 MHz, $CDCl_3$) ppm (δ): 189.93, 160.18, 158.46, 144.96, 138.80, 128.13, 128.05, 127.08, 126.38, 120.10, 118.45, 110.17, 104.06, 56.45 (OCH_3), 56.08 (OCH_3), 17.61 (SCH_3), 14.83 (SCH_3). LRMS (ES+) 390.83 $[M^+]$

Supplementary Figures

THE SYNTHESIS OF NOVEL THIODERIVATIVE CHALCONES AND THEIR INFLUENCE ON NF-κB, STAT3 AND Nrf2 SIGNALING PATHWAYS IN COLORECTAL CANCER CELLS

Katarzyna Papierska, Violetta Krajka-Kuźniak, Robert Kleszcz,
Tomasz Stefański, Rafał Kurczab, Maciej Kubicki

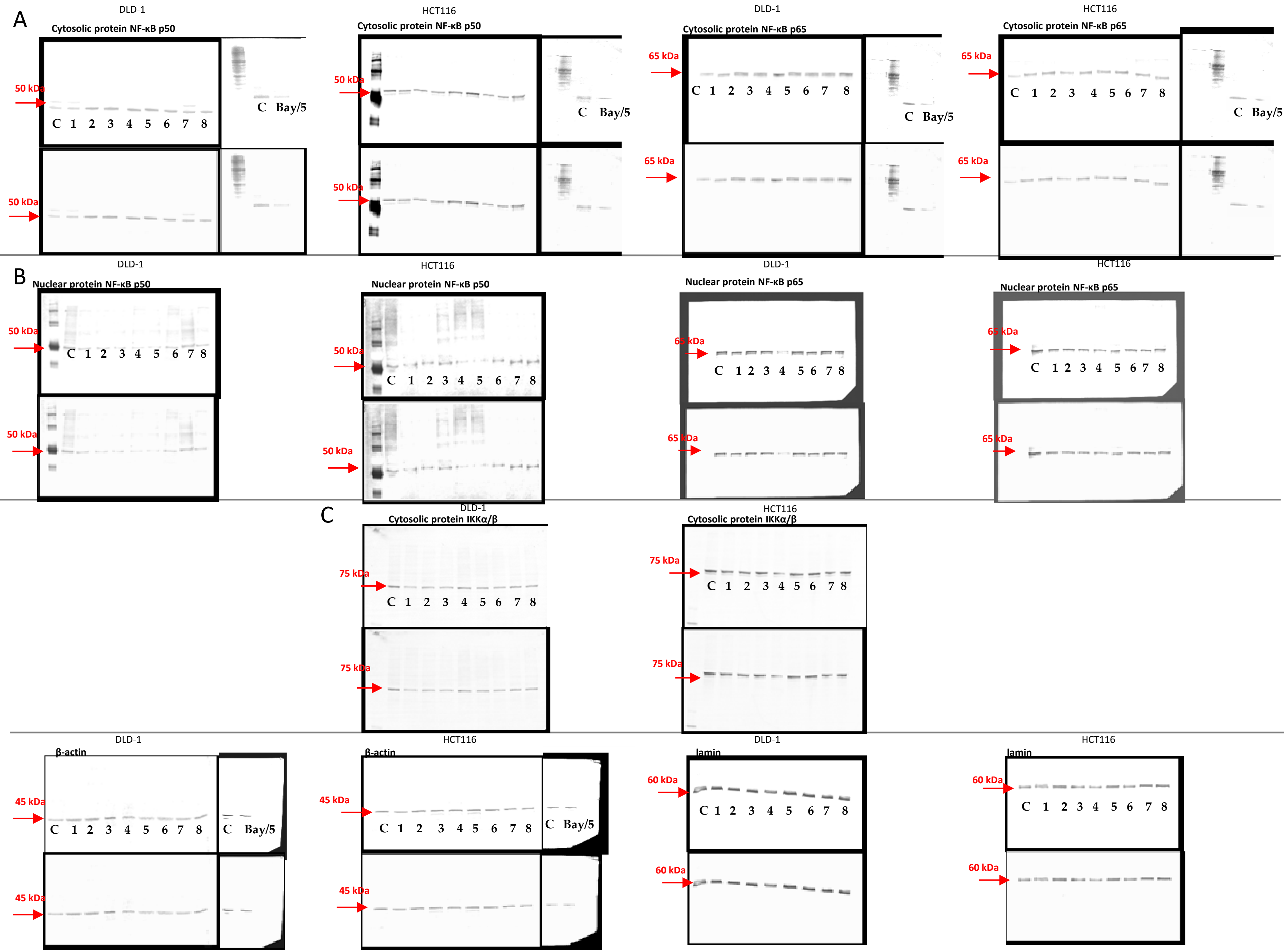
A
DLD-1
Cytosolic protein NF-κB p50
50 kDa
C 1 2 3 4 5 6 7 8
C Bay/5
HCT116
Cytosolic protein NF-κB p50
50 kDa
C Bay/5
DLD-1
Cytosolic protein NF-κB p65
65 kDa
C 1 2 3 4 5 6 7 8
C Bay/5
HCT116
Cytosolic protein NF-κB p65
65 kDa
C 1 2 3 4 5 6 7 8
C Bay/5
B
DLD-1
Nuclear protein NF-κB p50
50 kDa
C 1 2 3 4 5 6 7 8
HCT116
Nuclear protein NF-κB p50
50 kDa
C 1 2 3 4 5 6 7 8
DLD-1
Nuclear protein NF-κB p65
65 kDa
C 1 2 3 4 5 6 7 8
HCT116
Nuclear protein NF-κB p65
65 kDa
C 1 2 3 4 5 6 7 8
C
DLD-1
Cytosolic protein IKKα/β
75 kDa
C 1 2 3 4 5 6 7 8
HCT116
Cytosolic protein IKKα/β
75 kDa
C 1 2 3 4 5 6 7 8
DLD-1
β-actin
45 kDa
C 1 2 3 4 5 6 7 8
C Bay/5
HCT116
β-actin
45 kDa
C 1 2 3 4 5 6 7 8
C Bay/5
DLD-1
lamin
60 kDa
C 1 2 3 4 5 6 7 8
HCT116
lamin
60 kDa
C 1 2 3 4 5 6 7 8

Supplementary Figure F1. The representative immunoblots and their multiple exposure images respectively for Fig. 2 *The effect of synthetic thioderivative chalcone compounds on the level of NF-κB activation in DLD-1 and HCT116 cells.* **Panel A** - The levels of NF-κB, p50, and p65 protein in the cytosolic fraction. **Panel B** - The levels of NF-κB p50 and p65 protein in the nuclear fraction. **Panel C** - The levels of NF-κB, p50, and p65 protein in the cytosolic fraction from cells treated by Bay-117082/5. Data were normalized against the level of β-actin (cytosolic proteins) or lamin (nuclear proteins). **C - control; 1 – 1/1; 2 – 2/5; 3 – 3/5; 4 – 4/1; 5 – 5/5**; **6 – 6/5; 7 – 7/5; 8 – 8/5; Bay/5 – Bay-11-7082/5.**

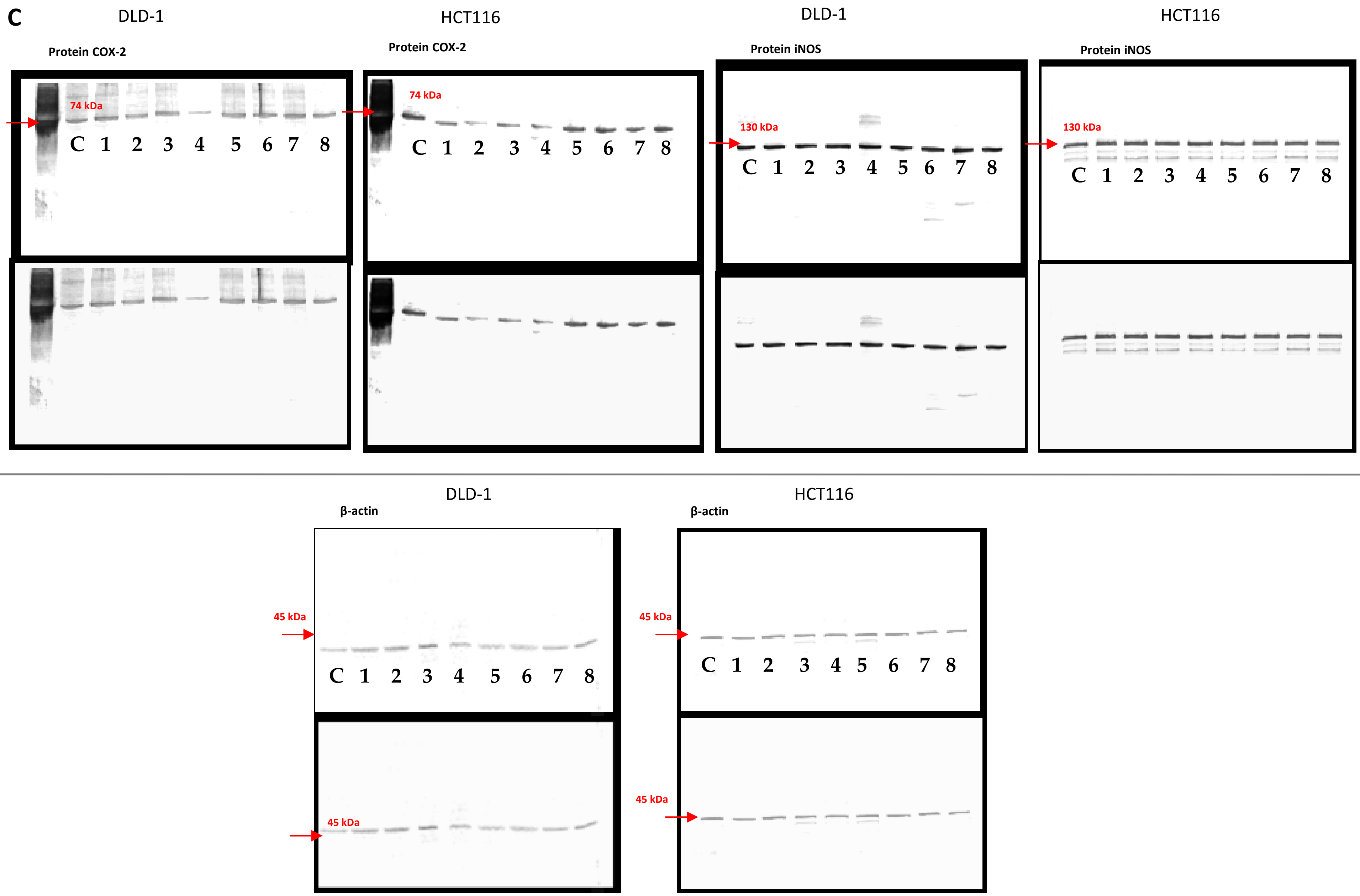

Supplementary Figure F2. The representative immunoblots and their multiple exposure images respectively for Fig. 3 *The effect of synthetic thioderivative chalcone compounds on the level of NF-κB expression in DLD-1 and HCT116 cells*. **Panel C** - The levels of COX-2 and iNOS proteins in the cytosolic fraction. Data were normalized against the level of β-actin. **C – control; 1 – 1/1; 2 – 2/5; 3 – 3/5; 4 – 4/1; 5 – 5/5**; **6 – 6/5; 7 – 7/5; 8 – 8/5.**

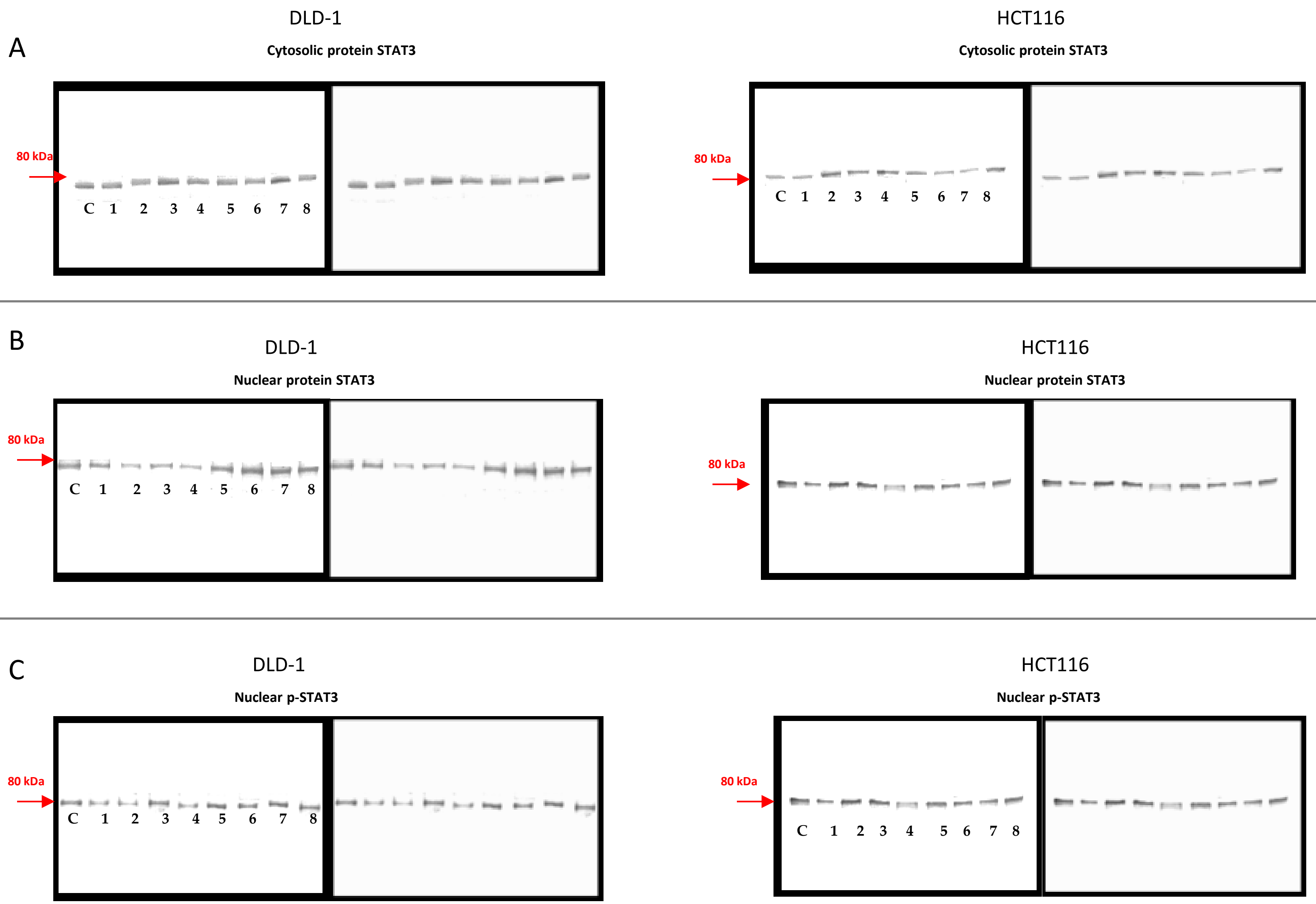
A
DLD-1
Cytosolic protein STAT3
80 kDa
C 1 2 3 4 5 6 7 8
HCT116
Cytosolic protein STAT3
80 kDa
C 1 2 3 4 5 6 7 8
B
DLD-1
Nuclear protein STAT3
80 kDa
C 1 2 3 4 5 6 7 8
HCT116
Nuclear protein STAT3
80 kDa
C
DLD-1
Nuclear p-STAT3
80 kDa
C 1 2 3 4 5 6 7 8
HCT116
Nuclear p-STAT3
80 kDa
C 1 2 3 4 5 6 7 8

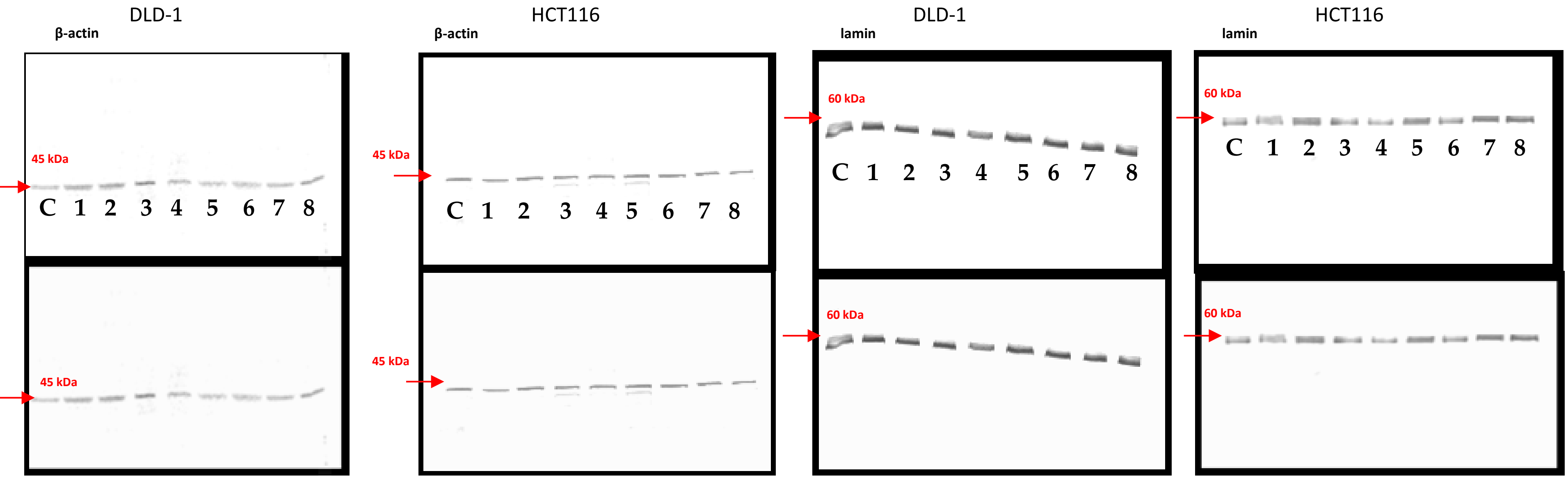

Supplementary Figure F3. The representative immunoblots and their multiple exposure images respectively for Fig. 4 *The effect of synthetic thioderivative chalcone compounds on the level of STAT3 activation in DLD-1 and HCT116 cells*. **Panel A** - The level of STAT3 protein in the cytosolic fraction. **Panel B** - The level of STAT3 protein in the nuclear fraction. **Panel C** - The level of p-STAT3 protein in the nuclear fraction. Data were normalized against the level of β-actin (cytosolic proteins) or lamin (nuclear proteins). **C – control; 1 – 1/1; 2 – 2/5; 3 – 3/5; 4 – 4/1; 5 – 5/5; 6 – 6/5; 7 – 7/5; 8 – 8/5.**

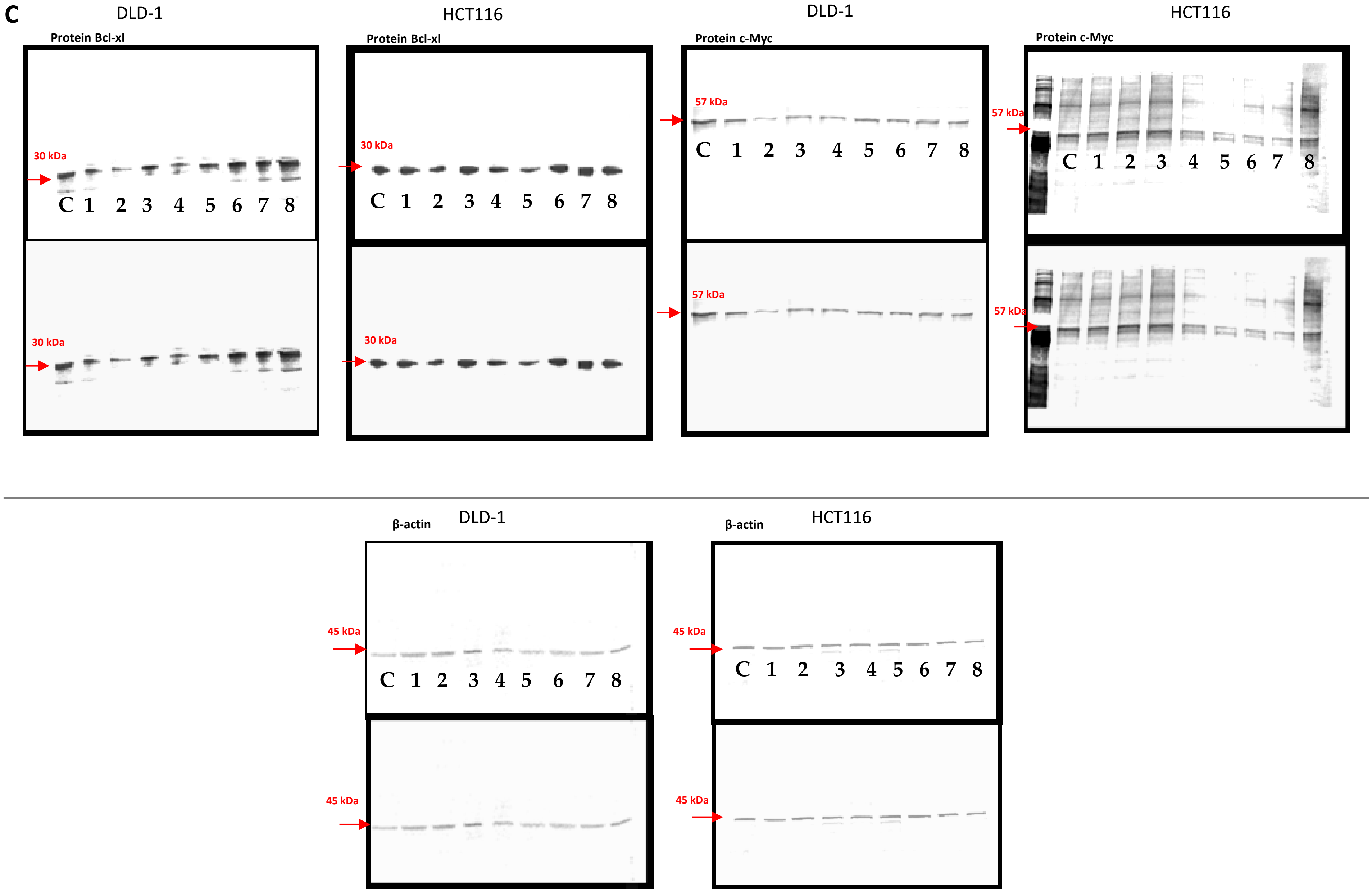

Supplementary Figure F4. The representative immunoblots and their multiple exposure images respectively for Fig. 5 *The effect of synthetic thioderivative chalcone compounds on STAT3 expression in DLD-1 and HCT116 cells.* **Panel C** - The levels of Bcl-xl and c-Myc protein in the cytosolic fraction. Data were normalized against the level of β-actin. **C – control; 1 – 1/1; 2 – 2/5; 3 – 3/5; 4 – 4/1; 5 – 5/5; 6 – 6/5; 7 – 7/5; 8 – 8/5.**

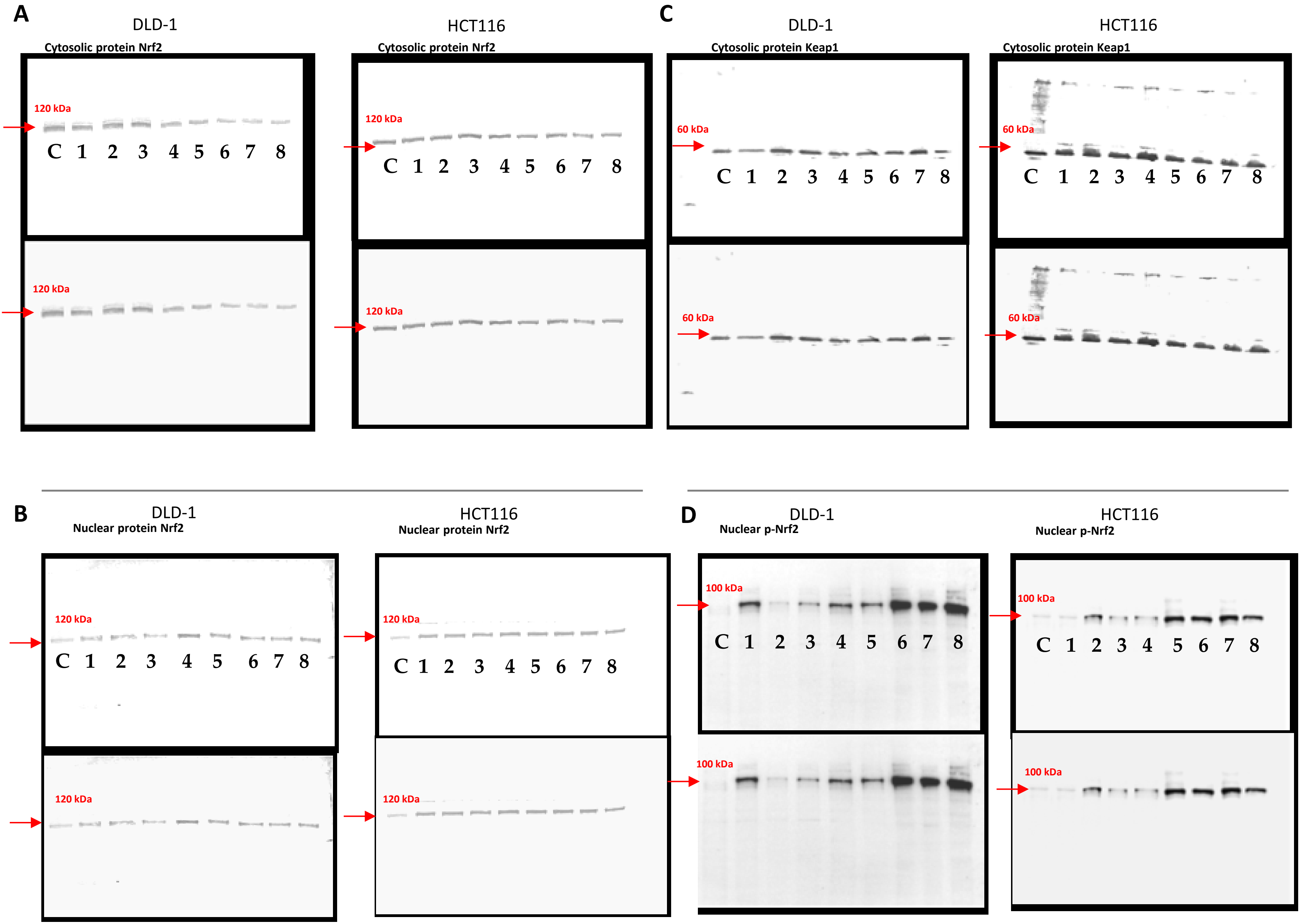

A
DLD-1
Cytosolic protein Nrf2
120 kDa
C 1 2 3 4 5 6 7 8
120 kDa
HCT116
Cytosolic protein Nrf2
120 kDa
C 1 2 3 4 5 6 7 8
120 kDa
C
DLD-1
Cytosolic protein Keap1
60 kDa
C 1 2 3 4 5 6 7 8
60 kDa
HCT116
Cytosolic protein Keap1
60 kDa
C 1 2 3 4 5 6 7 8
60 kDa
B
DLD-1
Nuclear protein Nrf2
120 kDa
C 1 2 3 4 5 6 7 8
120 kDa
HCT116
Nuclear protein Nrf2
120 kDa
C 1 2 3 4 5 6 7 8
120 kDa
D
DLD-1
Nuclear p-Nrf2
100 kDa
C 1 2 3 4 5 6 7 8
100 kDa
HCT116
Nuclear p-Nrf2
100 kDa
C 1 2 3 4 5 6 7 8
100 kDa

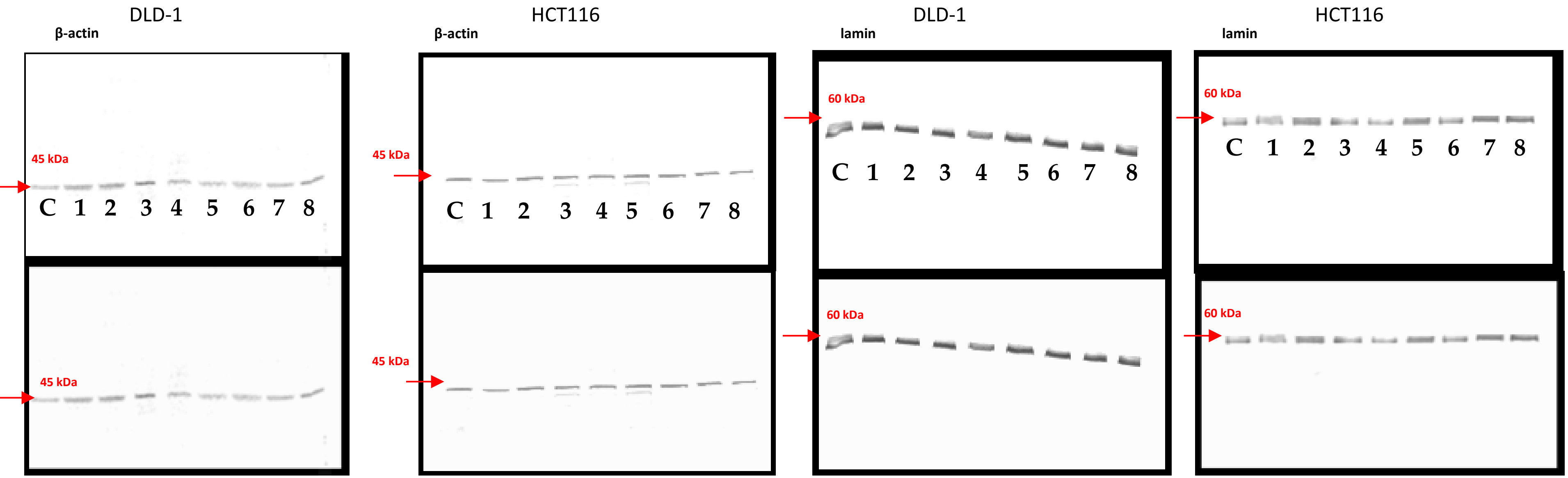

Supplementary Figure F5. The representative immunoblots and their multiple exposure images respectively for Fig. 6 *The effect of synthetic thioderivative chalcone compounds on Nrf2 activation in DLD-1 and HCT116 cells.* **Panel A** - The level of Nrf2 protein in the cytosolic fraction. **Panel B** - The level of Nrf2 protein in the nuclear fraction. **Panel C** - The level of Keap1 protein in the cytosolic fraction. **Panel D** - The level of p-Nrf2 protein in the nuclear fraction. Data were normalized against the level of β-actin (cytosolic proteins) or lamin (nuclear proteins). **C – control; 1 – 1/1; 2 – 2/5; 3 – 3/5; 4 – 4/1; 5 – 5/5; 6 – 6/5; 7 – 7/5; 8 – 8/5.**

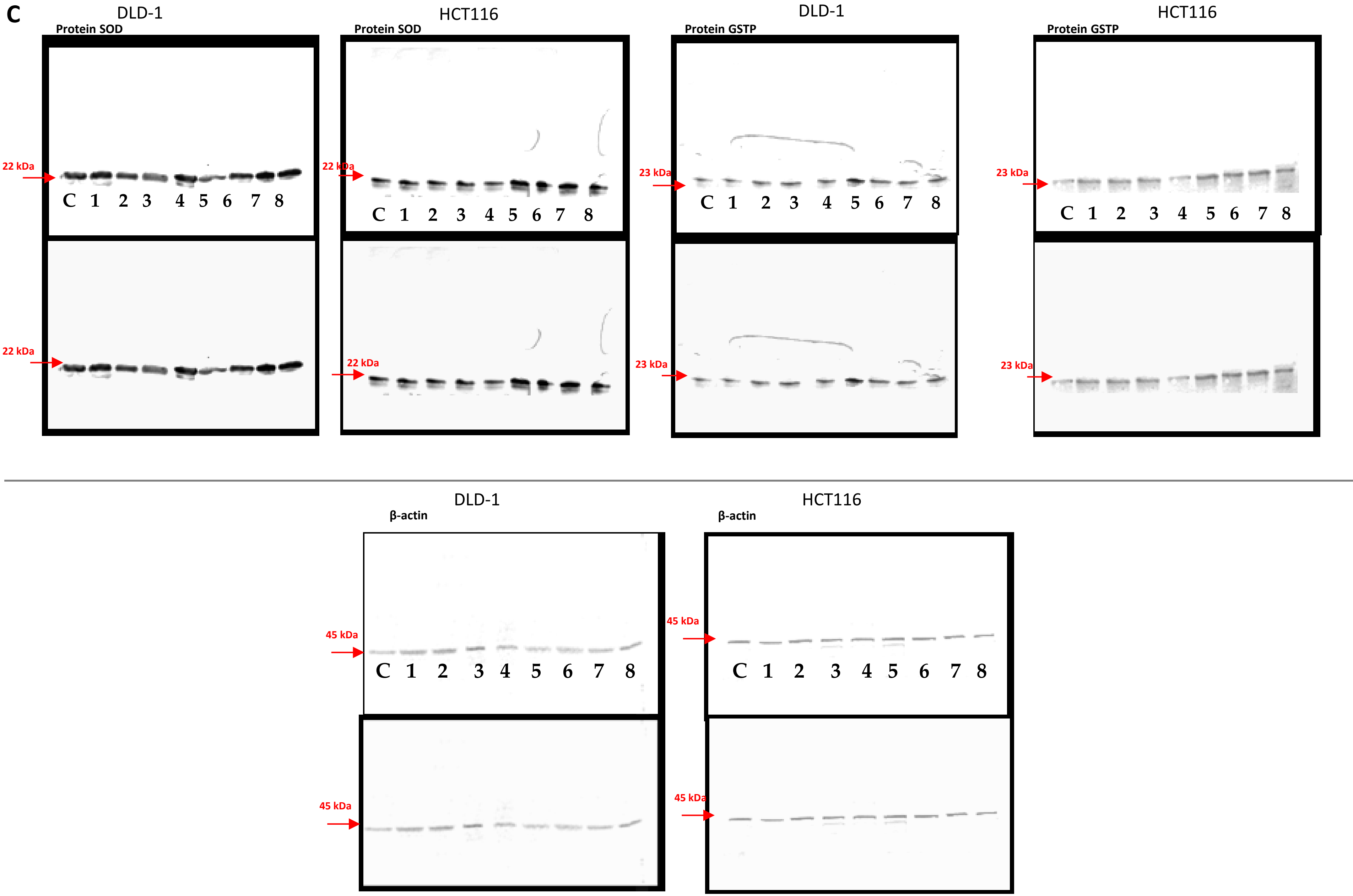

Supplementary Figure F6. The representative immunoblots and their multiple exposure images respectively for Fig. 7 *The effect of synthetic thioderivative chalcone compounds on Nrf2 expression in DLD-1 and HCT116 cells.* **Panel C** - The levels of SOD and GSTP proteins in the cytosolic fraction. Data were normalized against the level of β-actin. **C – control; 1 – 1/1; 2 – 2/5; 3 – 3/5; 4 – 4/1; 5 – 5/5; 6 – 6/5; 7 – 7/5; 8 – 8/5.**

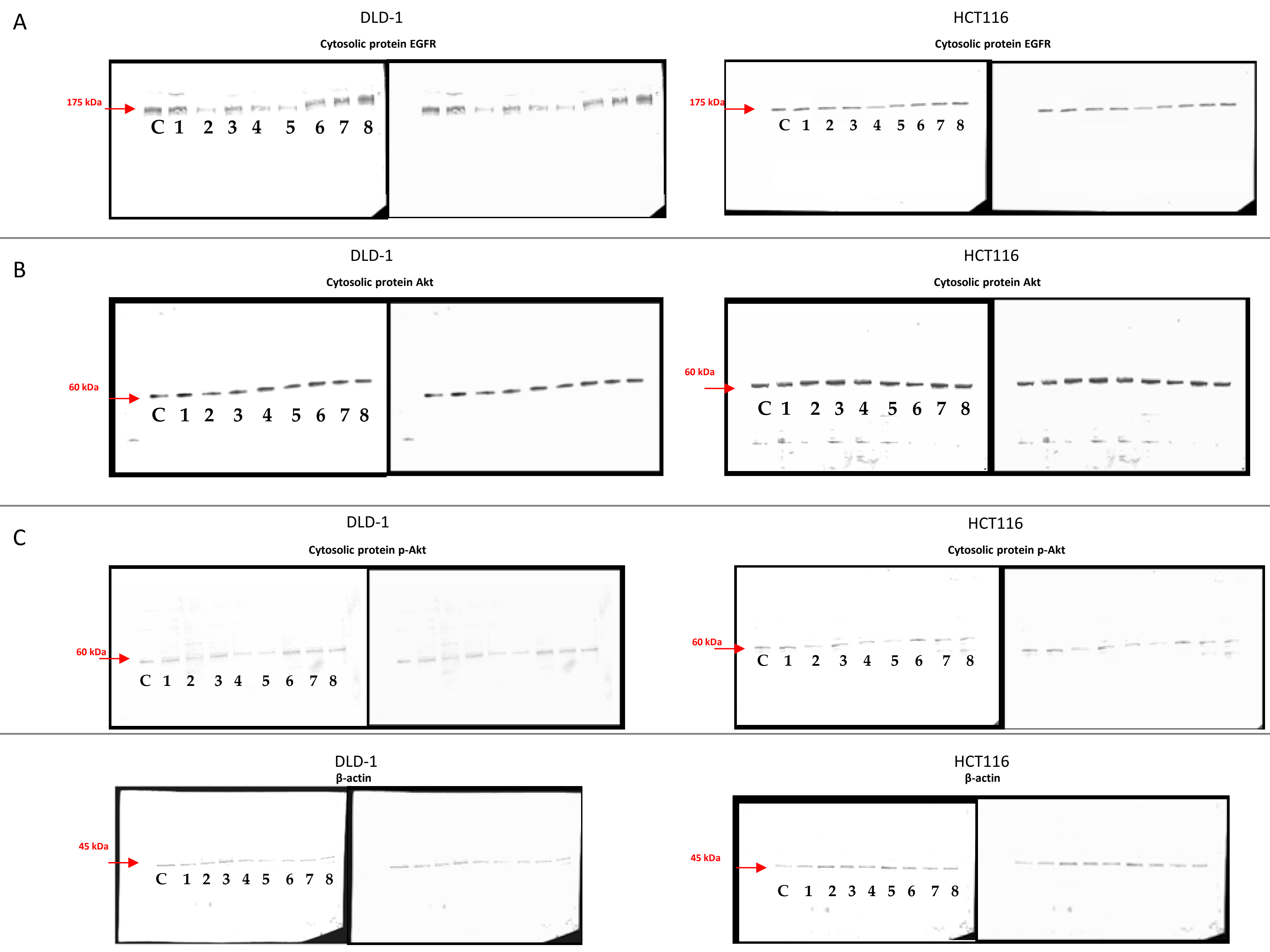
A
DLD-1
Cytosolic protein EGFR
175 kDa
C 1 2 3 4 5 6 7 8
HCT116
Cytosolic protein EGFR
175 kDa
C 1 2 3 4 5 6 7 8
B
DLD-1
Cytosolic protein Akt
60 kDa
C 1 2 3 4 5 6 7 8
HCT116
Cytosolic protein Akt
60 kDa
C 1 2 3 4 5 6 7 8
C
DLD-1
Cytosolic protein p-Akt
60 kDa
C 1 2 3 4 5 6 7 8
HCT116
Cytosolic protein p-Akt
60 kDa
C 1 2 3 4 5 6 7 8
DLD-1
β-actin
45 kDa
C 1 2 3 4 5 6 7 8
HCT116
β-actin
45 kDa
C 1 2 3 4 5 6 7 8

Supplementary Figure F7. The representative immunoblots and their multiple exposure images respectively for Fig. 8 *The effect of synthetic thioderivative chalcone compounds on the level of activation on EGFR activation in DLD-1 and HCT116 cells*. **Panel A** - The levels of EGFR protein in the cytosolic fraction. **Panel B** - The levels of Akt protein in the cytosolic fraction. **Panel C** - The levels of p-Akt protein in the cytosolic fraction. Data were normalized against the level of β-actin (cytosolic proteins). **C - control; 1 – 1/1; 2 – 2/5; 3 – 3/5; 4 – 4/1; 5 – 5/5**; **6 – 6/5; 7 – 7/5; 8 – 8/5.**

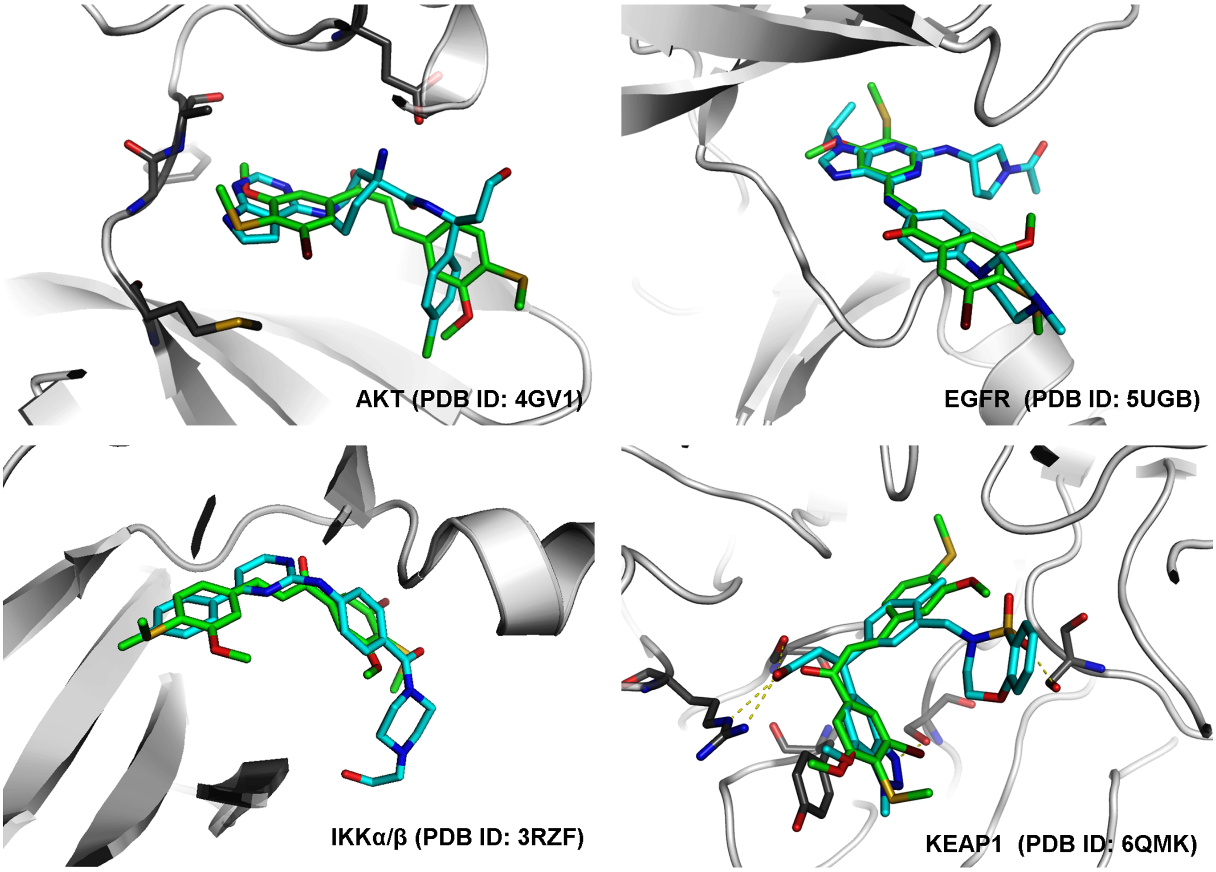

Supplementary Figure F8. Comparison of the binding mode of compounds **5** (green) with AKT inhibitor AZD5363 (PDB ID: 4GV1, IC50 = 4.2 nM); EGFR inhibitor PF-06747775 (PDB ID: 5UGB, IC50 = 161 nM); IKKα/β inhibitor (PDB ID: 3RZF), and Keap1 inhibitor (PDB ID: 6QMK, IC50 = 47 nM). IC50 values were extracted from BinidngDB. Illustrations were generated using PyMol software.
